# Dietary Polyphenols as Modulators of Redox Signalling: From the Antioxidant-Pro-Oxidant Continuum to Clinical Translation (2015–2025)

**DOI:** 10.3390/cimb48070732

**Published:** 2026-07-17

**Authors:** José Manuel Pérez de la Lastra, Celia María Curieses Andrés, Elena Bustamante Munguira, Celia Andrés Juan, Eduardo Pérez Lebeña

**Affiliations:** 1 Institute of Natural Products and Agrobiology, CSIC-Spanish Research Council, Avda. Astrofísico Fco. Sánchez, 3, 38206 San Cristobal de La Laguna, Spain; 2 Hospital Clínico Universitario de Valladolid, Avenida de Ramón y Cajal, 3, 47003 Valladolid, Spain; cmcuriesesa@saludcastillayleon.es (C.M.C.A.); ebustamante@saludcastillayleon.es (E.B.M.); 3 Cinquima Institute and Department of Organic Chemistry, Faculty of Sciences, Valladolid University, Paseo de Belén, 7, 47011 Valladolid, Spain; celia.andres.juan@uva.es; 4 Sistemas de Biotecnología y Recursos Naturales, 47625 Valladolid, Spain; info@glize.eu

**Keywords:** polyphenols, redox signalling, NRF2/Keap1, o-quinones, NQO1, hormesis, bioavailability, microbiota metabotypes, polyphenol oxidase, dose-response

## Abstract

Polyphenols often oscillate between antioxidant and pro-oxidant behaviours depending on structural motifs and context, yet the field still relies heavily on test-tube antioxidant assays that poorly predict cellular and clinical outcomes. This review (2015-2025) integrates chemistry, enzymology, and human data to frame polyphenols as modulators of redox signalling rather than mere radical scavengers. We first formalize the catechol/o-quinone-hydroquinone/p-quinone cycle and the role of NQO1 and Keap1/NRF2 thresholds. We then examine bioavailability, conjugation, and microbiota-derived metabolites (metabotypes), highlighting when the “active” species is a conjugate or a microbial derivative. We discuss safety through quinone speciation and adduct chemistry, and connect food processing (e.g., PPO-driven browning) with shifts in quinone pools. Contextual “levers” (pH, O_2_, Fe/Cu, oxidases) can flip antioxidant to pro-oxidant outputs, sometimes beneficial via hormesis and redox preconditioning. In humans, randomised trials and prospective cohorts point in a broadly consistent direction, although primary composite endpoints have often proved null, and observational associations should not be read as equivalent to trial evidence. Heterogeneous results across studies are largely explained by dose, adherence, metabotypes, matrix, and endpoint selection. We propose a practical dose-response framework and a reporting checklist to improve interpretation and translation. Recasting polyphenols as tuneable redox-signalling agents clarifies apparent contradictions across models and suggests precision-nutrition strategies (metabotype-aware) and food design approaches (PPO and quinone speciation) with potential in healthy ageing, muscle, and brain.

## 1. Introduction

Polyphenols entered nutrition science under the generous banner of “antioxidants”, a label that proved useful in food chemistry yet increasingly misleading in biology. Scavenging radicals in a cuvette is not the same as improving cellular resilience, and the last decade has clarified why: most benefits attributed to polyphenols emerge not from wholesale quenching of oxidants, but from the modulation of redox-sensitive signalling.

At physiologic pH and oxygen, catechol and hydroquinone motifs can ascend to o- and p-quinones; that chemistry is reversible in cells because it is embedded in an enzyme-guarded loop. NQO1 provides two-electron “rescue” of quinones, glutathione systems buffer soft electrophiles, and conjugation by UDP-glucuronosyltransferases (UGTs), sulphotransferases (SULTs), and catechol-O-methyltransferase (COMT) redirects products toward clearance. Meanwhile, low-amplitude hydrogen peroxide, generated directly or via enzyme systems, moves through peroxiporin channels and is decoded by redox relays to adjust protein tyrosine phosphatases and the Keap1-NRF2 axis. When these inputs are brief and buffers intact, the net effect is hormetic: a lean, adaptive strengthening of endogenous defences.

This mechanistic reframing forces attention to context. Processing steps and polyphenol oxidase (PPO) can shunt phenolics toward transient, recyclable quinones or into long-lived pigments and adducts that look potent in chemical assays yet do little *in vivo*.

**Figure 1:**
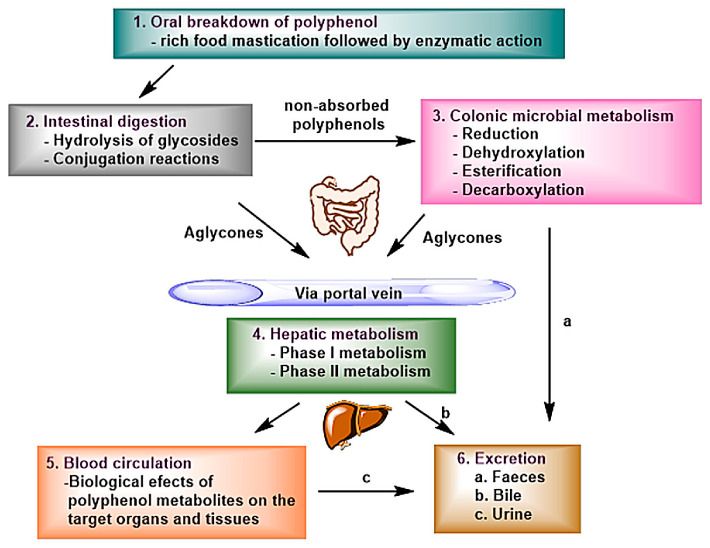
Absorption pathways and metabolism of dietary polyphenols, from oral breakdown and intestinal digestion through colonic microbial metabolism, hepatic processing, and eventual circulation, excretion, or biological action on target organs and tissues. Lower-case letters denote the three excretion routes shown in box 6: (a) faecal excretion of non-absorbed and colonic-derived metabolites, (b) biliary excretion following hepatic metabolism, and (c) urinary excretion following systemic circulation.

After ingestion, first-pass metabolism ensures that conjugates, rather than aglycones, dominate plasma. In the colon, microbes complete transformations that the host cannot, generating relatively stable “signalling metabolites” such as urolithins and phenyl-γ-valerolactones. Interindividual metabotypes, organ function, metal availability, and timing relative to meals or exercise together decide whether a given dose lands inside a family-specific “effective window” (Figure 1).

The human evidence mirrors this nuance. Large randomized trials and cohort studies now support vascular and neurocognitive benefits under realistic intakes, while also explaining null results when dose, adherence, matrix, or endpoints are poorly matched to mechanism. Against this backdrop, and building on the mechanistic and chemical foundations established in our 2023 work [1], the present review addresses a distinct question, namely under what real-world conditions of bioavailability, metabolism, food processing, and dose polyphenols actually translate into measurable effects on human health. We move deliberately from chemistry to cell signalling, through metabolism and microbiota, into food processing, epidemiology, and clinical trials, and finally to dose-response, interactions, and applications in healthy ageing.

Along the way, we emphasize measurement that matters (exposure biomarkers over intake surrogates), define a practical safety window, and propose shared resources, physicochemical data, reproducible scripts, and mechanistic maps, to make the field more cumulative. Our goal is simple: replace slogans with specifications, so that polyphenols can be designed and deployed as tuneable inputs to conserved stress-response networks.

## 2. Scope and Structure of the Review

This review is a focused, narrative synthesis that examines developments in polyphenol redox biology reported between 2015 and 2025, emphasizing mechanisms that plausibly operate at realistic dietary exposures. Section 2.1 sets out how the relevant literature was identified, and Section 2.2 explains how this synthesis relates to our earlier account. We privilege evidence that links chemistry to phenotype: the catechol/o-quinone and hydroquinone/p-quinone cycles, two-electron “rescue” by NQO1, glutathione economy and conjugation via UGTs, SULTs, and COMT, peroxiporin-mediated hydrogen-peroxide signalling, and the Keap1-NRF2 axis as a central integrator. Because circulating species are rarely aglycones, we foreground conjugates and microbiota-derived metabolites (notably urolithins and phenyl-γ-valerolactones) and treat food processing, especially PPO activity and oxygen/metal management, as first-order determinants of what biology ultimately “sees”.

We draw on peer-reviewed studies that report mechanistic readouts (NRF2 targets, thiol redox, peroxiredoxin states, endothelial function), objective exposure biomarkers (plasma/urine conjugates or microbial products), or hard clinical endpoints when available. We reference chemical assays (DPPH, FRAP, ORAC, CUPRAC; DPPH, 2,2-diphenyl-1-picrylhydrazyl; FRAP, ferric reducing antioxidant power; ORAC, oxygen radical absorbance capacity; CUPRAC, cupric reducing antioxidant capacity) only to interpret why they often diverge from *in vivo* behaviour. The review is not a systematic meta-analysis; instead, it aims to assemble an operational map, identify conditions that flip sign (pH, O_2_, labile Fe/Cu, matrix), and articulate testable predictions for intervention design.

Section 3 refines operational definitions and the mechanistic core of redox signalling. Section 4 examines bioavailability and conjugation. Section 5 addresses microbial transformation and metabotypes. Section 6 considers quinone chemistry, adducts, and safety. Section 7 and Section 8 connect processing to human evidence, clarifying why large-N studies sometimes read null. Section 9 develops dose-response “windows” and preconditioning logic. Section 10 translates these insights into precision-nutrition and product-design levers. Section 11 outlines controversies and open questions, and Section 12 distils practical conclusions. Throughout, we favour specificity, matrix, dose, timing, and readout, over generic “antioxidant” claims, aiming to replace slogans with specifications that can be implemented and falsified.

### 2.1. Literature Search Strategy

Literature was identified via PubMed, Web of Science Core Collection, and Scopus, covering January 2015 to December 2025, while a small number of foundational pre-2015 studies were retained for mechanistic continuity. This search was complemented by forward and backward citation tracking from key articles identified during the initial screening. Searches combined controlled vocabulary, including Medical Subject Headings such as “Polyphenols”, “Oxidative Stress”, and “NF-E2-Related Factor 2”, with free-text terms covering polyphenol, flavonoid, catechol, quinone, NRF2, Keap1, NQO1, hydrogen peroxide, hormesis, bioavailability, glucuronidation, sulphation, gut microbiota, urolithin, phenyl-valerolactone, polyphenol oxidase, and clinical trial, joined through Boolean operators and truncation where appropriate. Eligible sources comprised peer-reviewed original research and review articles published in English that reported mechanistic, biochemical, or clinical data relevant to polyphenol redox chemistry, bioavailability, microbial metabolism, food processing, or human health outcomes. Conference abstracts, non-peer-reviewed preprints, and studies reporting chemical antioxidant assays without any biological or clinical readout were generally excluded from detailed discussion, although a limited number are referenced briefly to illustrate the shortcomings of such assays. Titles and abstracts were screened by one author for relevance, with any ambiguous or borderline records checked against the full text before a decision was made. The resulting reference list was then reviewed by all co-authors, who confirmed topical coverage and mechanistic accuracy before the final selection was agreed.

### 2.2. Novelty Relative to Our Previous 2023 Review

This review builds directly upon our earlier work, which examined polyphenols primarily as antioxidant and pro-oxidant compounds, using three representative molecules, resveratrol, hydroxytyrosol, and luteolin, to illustrate the reversible oxidation of the catechol group to its corresponding o-quinone [1]. That earlier account centred on the electron and proton donating chemistry of individual polyphenols, on their capacity to chelate metals, and on their influence over the Keap1-NRF2-ARE axis, together with a set of transcription factors and enzymes relevant to cancer biology.

The present review extends that framework in four distinct directions. First, rather than examining a small number of representative compounds, it treats bioavailability, conjugation, and microbiota-derived metabolism as first-order determinants of which chemical species the body actually encounters after ingestion, a dimension that received little attention in the earlier work. Second, it incorporates food processing and polyphenol oxidase activity as variables that shape quinone speciation well before ingestion occurs, connecting food chemistry directly to the redox signalling later observed in cells. Third, it synthesises the clinical and epidemiological literature published between 2015 and 2025, including large randomised trials and cohort studies, in order to explain why human evidence has often appeared heterogeneous or null, an analysis that fell outside the scope of the earlier review. Fourth, it proposes a practical dose-response framework and a reporting checklist intended to guide future study design, moving beyond the earlier focus on mechanism alone towards translational application.

Read together, the two reviews should be seen as complementary rather than overlapping. The 2023 work established the chemical and enzymatic basis for understanding polyphenols as ambivalent antioxidant and pro-oxidant agents. The present review builds on that foundation to ask a different question, namely under what real-world conditions of bioavailability, metabolism, matrix, and dose these compounds actually influence human health, and how future research and food design might account for that context.

## 3. Updated Conceptual Framework of the Antioxidant-Pro-Oxidant Continuum

Here, we recast the antioxidant-pro-oxidant “continuum” as a dynamic system rather than a binary label. What cells experience at any moment is the balance between the rate at which oxidizing and electrophilic equivalents are introduced and the capacity of local buffers and enzymes to absorb, repair, or export them.

Polyphenols fit naturally into this scheme because catechol and hydroquinone motifs feed reversible pools of o- and p-quinones whose fate is context-dependent: in a well-buffered compartment with intact two-electron repair, they cycle quietly and bias adaptive programs; in metal-rich, thiol-poor niches, the same pools amplify peroxide flux and covalent pressure. Benefit and harm therefore separate less by molecular identity than by intensity, timing, and compartmentalization, precisely the conditions that give rise to hormesis, where small, recoverable perturbations yield a net increase in resilience [2].

Translating that logic into practice requires explicit operationalization. Throughout this section, we anchor interpretation to defined matrices (buffer, food, plasma, cytosol), timescales (seconds to hours for signalling, days to weeks for adaptation), and readouts that map onto mechanisms rather than surrogates. Low-amplitude H_2_O_2_ transients, often shaped by peroxiporins and redox relays, are treated as signals that modulate protein tyrosine phosphatases and the Keap1-NRF2 sensor, not as damage *per se* [3].

Quinone traffic is evaluated against the capacity of NQO1 to enforce two-electron “rescue”, and of glutathione S-transferase GST/UGT/SULT systems and transporters to neutralize and clear products. With those guardrails in place, the subsections that follow set out (i) operational definitions that avoid categorical traps, (ii) the reversible catechol/quinone circuitry and NQO1’s gatekeeping role, (iii) NRF2/ARE threshold behaviour, and (iv) the environmental levers, pH, oxygen tension, labile Fe/Cu, and oxidases, that flip the sign of the response [4].

### 3.1. Operational Definitions: Antioxidant vs. Pro-Oxidant and Why Context Rules

An “antioxidant” in this review is defined operationally, not metaphysically: it is any agent that, in a specified system and timeframe, diminishes net oxidative damage or lowers the steady-state level of reactive species. That reduction may occur through rapid radical scavenging, metal chelation, triplet quenching, or, crucially in living systems, by inducing endogenous defences such as the glutathione system or the NRF2-ARE pathway [5].

A “pro-oxidant”, by contrast, is any agent that, under the same explicit conditions, increases net oxidative flux or provokes oxidative lesions, whether by redox cycling with molecular oxygen, reducing Fe^3+^/Cu^2+^ into Fenton-active forms, or generating electrophilic intermediates that deplete cellular thiols. These definitions are intentionally outcome-based because identical molecules can swap roles when the context changes [6].

Context rules for three reasons. First, chemistry is conditional: pH, oxygen tension, and the presence of trace metals shift redox potentials and reaction rates. Catechol-type polyphenols that efficiently intercept radicals at low micromolar levels may, at higher concentrations or in metal-rich media, autoxidize to quinones and produce hydrogen peroxide [7].

Second, biology is buffered and compartmentalized. The same quinone that appears pro-oxidant in a cell-free assay may be neutralized or recycled by cellular NADPH, glutathione, and enzymes such as NQO1, and may even act indirectly as a signalling cue that raises antioxidant capacity. The result is hormesis: a dose range where mild, transient “pro-oxidant” pressure yields a net antioxidant phenotype by preconditioning defences [8].

Third, time matters. Early bursts of oxidants can be followed by longer-lived adaptive responses; whether an intervention is labelled antioxidant or pro-oxidant depends on when one measures and what one chooses to measure [9].

Accordingly, classification should be tied to clearly defined matrices (buffer, food, plasma, cytosol), explicit readouts (radical decay, H_2_O_2_ flux, protein thiol status, DNA damage), and realistic dosing. Framed this way, polyphenols cease to be paradoxical: they are modulators of redox signalling whose apparent contradictions mostly reflect shifts in environment, concentration, and timescale [10].

### 3.2. The Catechol ⇄ o-Quinone/Hydroquinone ⇄ p-Quinone Cycle and Its Reversibility: Role of NQO1 in 2e^−^ Reduction

**Figure 2:**
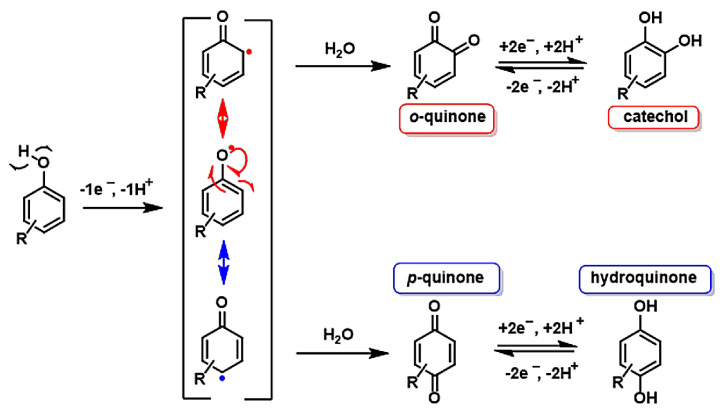
Oxidation mechanisms of catechol and hydroquinone, showing the redox process of catechol to o-quinone and hydroquinone to p-quinone through the loss or acceptance of two protons (2H^+^) and two electrons (2e^−^).

At the core of many polyphenols lies a deceptively simple redox couple. Catechols oxidize to o-quinones, and hydroquinones to p-quinones, through one-electron steps that pass via semiquinone radicals or through concerted two-electron transfers (Figure 2).

In aqueous, oxygenated media, the one-electron route is notoriously leaky: semiquinones reduce O_2_ to superoxide, which dismutates to hydrogen peroxide, seeding secondary oxidations. Quinones thus formed are electrophiles that undergo Michael addition with cysteine and other nucleophiles, depleting glutathione and modifying proteins at redox-sensitive thiols. Yet the same couples are, in principle, reversible [2].

The extent of reversibility depends on substitution patterns that set bond dissociation energies, the local pH (which controls phenolate formation), and the availability of reducing equivalents. In buffered biological compartments, catechol/quinone pairs do not drift freely toward damage, they are embedded in enzyme-guarded circuits that can either short-circuit redox cycling or, if overwhelmed, amplify it [11].

**Figure 3:**
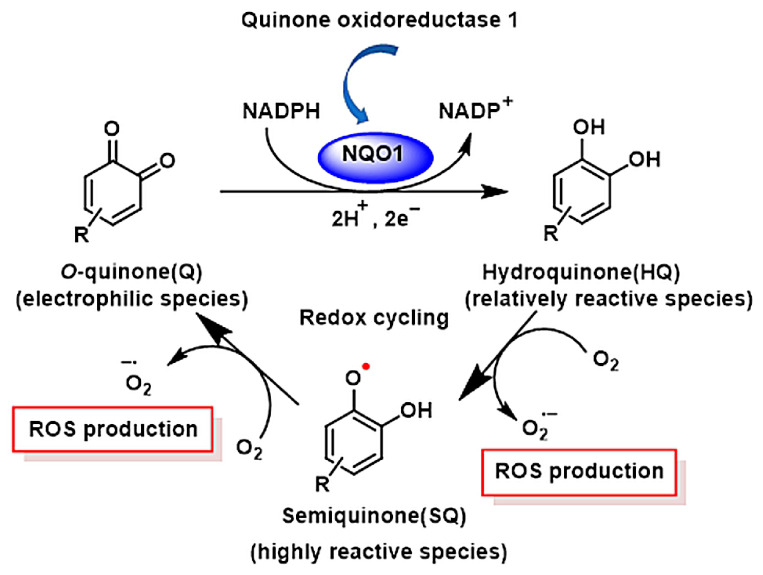
NQO1 catalyses the two-electron reduction of quinones to hydroquinones, which is commonly proposed as a detoxification mechanism. Quinones, which depend on their structure, are electrophilic species capable of reacting with cellular nucleophiles, and two-electron reduction removes a reactive electrophile from a biological system. Furthermore, two-electron reduction prevents the formation of semiquinones, which, depending on the redox potential, can interact with molecular oxygen to generate ROS capable of inducing cellular damage.

NQO1 (DT-diaphorase) is pivotal in this regard. This flavoprotein catalyses obligate two-electron reduction of quinones to hydroquinones using NAD(P)H, deliberately bypassing the semiquinone state. By doing so, NQO1 suppresses the single-electron back-reactions with oxygen that generate reactive oxygen species, and it temporarily lowers electrophilicity, giving cells a window to conjugate or export the reduced products. In contexts where NQO1 is abundant, often after NRF2 activation, the cycle closes neatly: quinones are pulled back to hydroquinones and then steered toward phase II metabolism (e.g., glucuronidation, sulphation) or sequestration. The net effect is cytoprotection, because both oxidative flux and covalent protein stress are blunted (Figure 3) [12].

The degree of protection NQO1 confers is substrate-dependent rather than uniform across every quinone. For the catechol-derived quinones typical of dietary polyphenols, the resulting hydroquinones tend to be comparatively stable and resist spontaneous re-oxidation, so two-electron reduction closes the cycle efficiently. For other quinone structures, including several naphthoquinones and some strongly oxidising quinone methides, the hydroquinone product can retain a low redox potential and re-oxidise rapidly once oxygen is available. In those specific cases, NQO1 reduction alone does not arrest redox cycling and can even contribute to bioactivation rather than detoxification, which is why the protective role of NQO1 should be read as conditional on the chemistry of the quinone in question.

**Figure 4:**
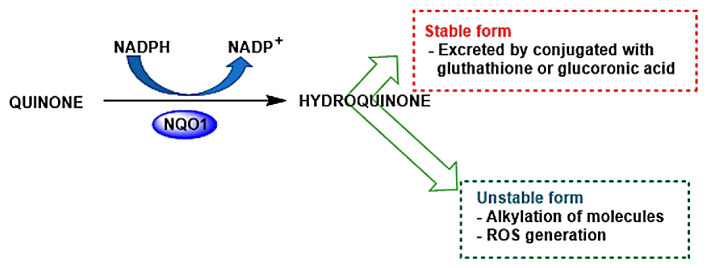
Hydroquinone formed by the reduction of quinone may be stable or unstable, depending on its structure, and this stability determines whether it is safely conjugated or instead re-oxidised to regenerate a reactive quinone species.

The very same chemistry, however, can flip sign. If quinone production outpaces NQO1 capacity, or if the reduced products are unstable and rapidly re-oxidize in the presence of oxygen or redox-active metals, a futile cycle emerges: repeated reduction and autoxidation generate peroxide continuously while siphoning NAD(P)H and glutathione (Figure 4) [13].

Under such load, electrophile-driven protein adduction and thiol depletion dominate, tilting the outcome toward cytotoxicity. The catechol/quinone system is therefore best understood as a controllable loop whose biological meaning is written not in the molecule alone, but in the balance between formation of quinones, enzymatic two-electron repair by NQO1, and the surrounding capacity to neutralize or export the reduced species [14].

### 3.3. NRF2/ARE Signalling Thresholds and the Keap1 Sensor

NRF2 is best understood as a fast-acting rheostat for cytoprotective capacity rather than a simple on/off switch. Under basal conditions, the adaptor Keap1 recruits NRF2 to a CUL3-based E3 ligase, targeting it for rapid ubiquitination and proteasomal degradation. The interaction follows a “hinge-and-latch” logic: a high-affinity ETGE motif on NRF2 anchors the complex, while a lower-affinity DLG motif licenses efficient turnover. What interrupts this cycle, and thereby raises NRF2 levels, is the chemical state of specific, highly nucleophilic cysteines on Keap1. Electrophiles and certain oxidants modify these thiols, loosening the ligase assembly, slowing NRF2 destruction, and allowing newly synthesized NRF2 to accumulate, translocate to the nucleus, dimerize with small Maf proteins, and engage antioxidant response elements (AREs) to drive genes such as NQO1, HO-1, GCLC/GCLM, and SLC7A11 [15].

Not all cysteines contribute equally, and not all stressors write the same “message” into Keap1. Cys151 in the BTB domain is a sensitive target for soft electrophiles; its modification perturbs CUL3 engagement. Cys273 and Cys288 within the intervening region (IVR) also act as key sensors, and distinct reagents show partial selectivity among these residues, a “cysteine code” that shapes the amplitude and duration of NRF2 activation [16].

Pure H_2_O_2_ only weakly and transiently affects Keap1 directly. In cells, H_2_O_2_ more often signals through redox relays (notably peroxiredoxins) that transmit oxidation to Keap1 or its partners. Electrophilic stress, by contrast, modifies Keap1 cysteines directly via Michael addition or related chemistry, typically at lower nominal doses [17].

Threshold behaviour emerges from this architecture. The effective set-point for NRF2 activation depends on Keap1 abundance and neddylation status of CUL3, the cellular thiol buffer (especially glutathione), the rate of Keap1 turnover and autophagic flux (p62/SQSTM1 competes for Keap1), and the concurrent induction of detoxifying enzymes [18].

Polyphenols intersect this network in two ways: by generating mild, localized H_2_O_2_ that engages redox relays, and by forming electrophilic quinones that modify Keap1 directly. Low, transient inputs tend to produce an adaptive NRF2 program: persistent or overwhelming inputs deplete thiols, saturate the relay, and blur adaptation into proteotoxic stress. Careful reporting of matrix, timing, and dose is therefore essential when labelling a stimulus as “NRF2-activating” versus injurious [19].

### 3.4. Factors That Tilt the Balance: pH, Dissolved O_2_, Free Fe/Cu, PPO and Other Oxidases (P450, COX-2, MAO, XO)

The redox behaviour of polyphenols is exquisitely sensitive to their surroundings, and small shifts in chemistry can upend biological outcomes. pH is the first lever. Many phenols become markedly more reactive upon deprotonation: the phenolate anion lowers the barrier to electron transfer and accelerates autoxidation to quinones [20].

Near neutral pH, catechols and gallates therefore oxidize faster than in acidic media, while O-methylation or lack of ortho-dihydroxylation blunts this effect. pH also governs metal binding: deprotonated oxygens coordinate Fe^3+^ and Cu^2+^ more avidly, which can either dampen redox cycling by sequestration or, if the complex remains labile, catalyse one-electron pathways that seed reactive oxygen species [21].

Oxygen availability is the second lever. Autoxidation rates scale with dissolved O_2_, and so does the fate of semiquinones. In well-oxygenated buffers or extracellular spaces, semiquinones efficiently reduce O_2_ to superoxide, which disproportionates to hydrogen peroxide; in compartments with lower O_2_ or robust reductive capacity, the same intermediates are steered toward two-electron repair [22].

This interplay becomes particularly consequential in the presence of redox-active iron and copper. Trace Fe^2+^/Fe^3+^ and Cu^+^/Cu^2+^ couple phenoxyl radicals to Fenton chemistry, converting otherwise modest oxidant fluxes into injurious hydroxyl equivalents and depleting cellular thiols. Conversely, tight chelation or protein sequestration of these metals raises the threshold for pro-oxidant cascades [23].

Enzymes add a further layer of control. Polyphenol oxidase (PPO) in plant tissues and foods catalyses the o-hydroxylation and subsequent two-electron oxidation of monophenols and catechols to o-quinones, effectively short-circuiting radical steps and rapidly building electrophile pools that drive browning and protein adduction. In mammalian systems, several oxidases intersect polyphenol chemistry. The peroxidase arm of cyclooxygenase (COX-2) and heme-peroxidases can one-electron oxidize phenolics to phenoxyl radicals in the presence of hydroperoxides [24].

Cytochrome P450 isoforms generate oxygenated metabolites and, under certain conditions, substrate radicals that feed back into redox cycles. Monoamine oxidase (MAO) yields hydrogen peroxide as a stoichiometric by-product, raising local oxidant tone, while xanthine oxidase (XO) produces superoxide and peroxide that can either be quenched by polyphenols or drive their oxidation. The net phenotype, antioxidant or pro-oxidant, thus emerges from a moving equilibrium between solution chemistry, metal speciation, oxygen supply, and enzyme activities that channel polyphenols toward protective signalling or toward electrophile- and ROS-driven stress [25].

**Figure 5:**
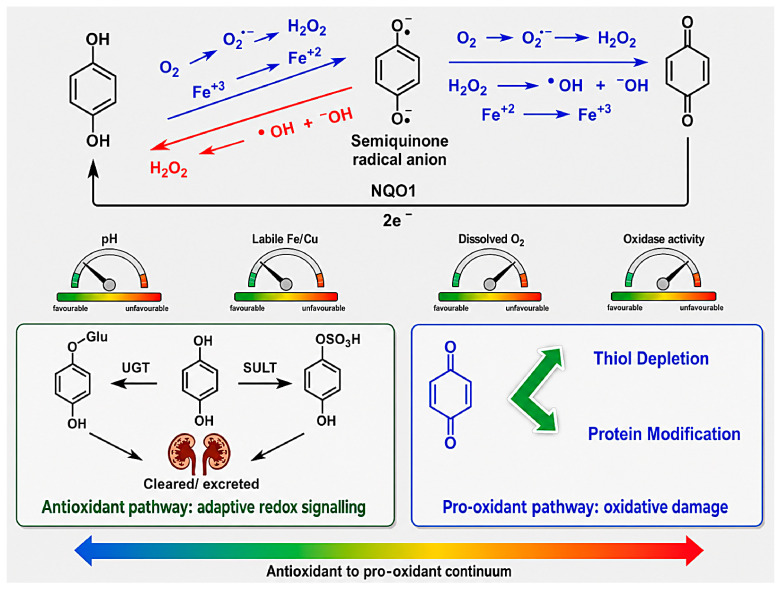
Schematic representation of how pH, dissolved oxygen, labile iron/copper and oxidase activity act together as a biological switch, determining whether catechols and hydroquinones are safely metabolised through NQO1 or instead driven toward cytotoxic quinone pathways.

To make this switching behaviour easier to grasp at a glance, Figure 5 brings together the four contextual levers just discussed, namely pH, dissolved oxygen, labile iron and copper, and oxidase activity, and shows how their combined state tips a given polyphenol toward antioxidant or pro-oxidant behaviour. Acidic conditions slow the generation of quinones, dissolved oxygen acts as the terminal electron acceptor that drives the initial oxidation of catechols to semiquinones, labile iron and copper catalyse the breakdown of hydrogen peroxide into aggressive hydroxyl radicals and form semiquinone complexes that accelerate oxidative damage, and enzymes such as polyphenol oxidase, cytochrome P450 and monoamine oxidase convert precursor phenols into reactive quinones. Rather than treating antioxidant and pro-oxidant character as a fixed property of the molecule itself, the figure places these four variables along independent axes and marks out the region where quinone traffic is cleared quietly through NQO1-mediated repair and conjugation, alongside the region where the same chemistry instead drains thiol pools and generates hydroxyl radicals through Fenton chemistry. The intention is that a reader can look up any combination of pH, oxygen tension, metal availability, and oxidase tone and read off which side of the continuum a given polyphenol is likely to occupy within that particular tissue or experimental system.

### 3.5. From “Scavenging” to Redox Signalling: H_2_O_2_ as a Second Messenger and Hormesis

The classic story casts polyphenols as radical sponges, mopping up chemical mischief before it touches biomolecules. That picture is tidy but incomplete. In living systems, the more decisive currency is not the instantaneous disappearance of a radical in a cuvette, but the orchestration of signals that recalibrate cellular defences [26].

H_2_O_2_ sits at the centre of this shift. At low, spatially confined levels, H_2_O_2_ behaves less like a toxin and more like a courier, transmitting information through selective, reversible oxidation of cysteine residues in proteins that control metabolism, stress responses, and gene expression [27].

This selectivity is not an accident. H_2_O_2_ seldom modifies targets directly; it is intercepted by high-affinity peroxiredoxins, which then pass the oxidative equivalent to partner proteins, a redox relay that affords both speed and specificity. Short-lived increases in H_2_O_2_ can transiently inactivate protein tyrosine phosphatases, amplifying kinase signalling, or adjust the activity of metabolic switches [28].

In parallel, electrophilic derivatives of polyphenols and H_2_O_2_-driven relays converge on the Keap1-NRF2 axis, easing NRF2 accumulation and transcription of genes that expand antioxidant capacity and repair. The geography matters too. Mitochondria and NADPH oxidase complexes generate H_2_O_2_ in defined microdomains, aquaporins facilitate its diffusion across membranes, yet buffering by thiols and peroxiredoxins constrains spread, preserving a local signal rather than a global insult [29].

Hormesis gives this biology its practical meaning. Modest, transient oxidative inputs, of the sort that polyphenols can help shape, provoke a measured adaptive program: induction of glutathione synthesis, upregulation of detoxifying enzymes, reinforcement of proteostasis, and adjustments in mitochondrial maintenance. Push harder or longer, and the same pathways buckle under thiol depletion and electrophile burden [30].

The difference between benefit and harm is therefore a window, not a line: set by dose, timing, metal availability, oxygen tension, and the existing tone of redox enzymes. Interventions that respect that window will rarely look dramatic in a single time point or in a chemical assay, but over physiological timescales, they deliver the outcome that matters most, greater resilience to stress [31].

## 4. Which Molecule Actually Acts *In Vivo*? Bioavailability and Metabolism

Before turning to mechanisms, it helps to ask which chemical entities the body actually “sees” after a polyphenol-rich meal. The answer is rarely the aglycones used *in vitro*. In the small intestine and liver, enterocyte and hepatocyte first-pass metabolism triages absorbed phenolics through rapid Phase II edits, glucuronidation and sulphation for most scaffolds, O-methylation for catechols, while membrane transporters (MRP2/3, BCRP, selected OATPs) shape how long those conjugates linger in tissues or recirculate via bile. In parallel, a substantial fraction escapes proximal uptake and meets the microbiota, which cleaves rings and dehydroxylates to yield smaller, more permeable “signalling metabolites” (e.g., urolithins, phenyl-γ-valerolactones, phenyl-acids) [32].

Local deconjugation by β-glucuronidases at inflamed or hypoxic mucosa can regenerate aglycones transiently, adding spatial nuance. The net exposure profile is therefore a slow-rise, low-amplitude mixture of conjugates and microbial products, modulated by matrix, transport, organ function, and metabotype, conditions that set the stage for the specific consequences of conjugation described next [33].

### 4.1. Slow Absorption, Conjugation (Glucuronides/Sulphates) and Mechanistic Consequences

Dietary polyphenols reach systemic circulation slowly and sparsely, a pharmacokinetic pattern that follows from how they are presented in foods and handled at epithelial barriers. Many arrive as glycosides or esterified forms that require hydrolysis in the intestinal lumen or at the brush border before the aglycone can even approach the membrane. Even then, uptake is modest and tightly coupled to first-pass metabolism in enterocytes and hepatocytes, where phenolic hydroxyls are rapidly masked by UDP-glucuronosyltransferases and sulphotransferases. The bloodstream therefore carries conjugates, mono- and diglucuronides, monosulphates, and mixed species, rather than the aglycones that dominate *in vitro* studies [34].

**Figure 6:**
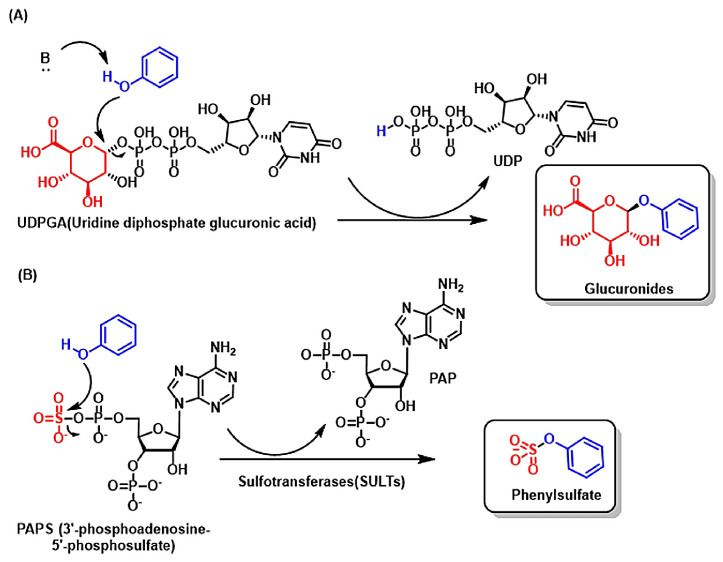
(**A**) Glucuronidation of phenols by nucleophilic attack on the carbon of UDP-glucuronic acid. Base B facilitates the abstraction of a proton from the phenol. (**B**) General mechanism of PAPS-dependent sulphotransferase.

Those chemical edits have consequences that are more than pharmacokinetic housekeeping. Glucuronidation removes hydrogen-donating phenolic OH groups and introduces bulky, polar moieties, while sulphation adds a dianionic handle. Both changes curb membrane permeability, dampen radical chemistry and metal chelation, and markedly reduce the tendency to autoxidize to electrophilic quinones (Figure 6) [35].

In short, conjugation steers polyphenols away from direct scavenging and toward roles defined by transport, compartmentalization, and controlled deconjugation. Conjugates bind to albumin and circulate longer at low concentrations; some are substrates for efflux and uptake transporters that shape tissue exposure, and, importantly, β-glucuronidase activity in inflamed or hypoxic microenvironments can regenerate aglycones locally, creating “on-site” pulses of reactivity that are invisible to bulk plasma measurements [36].

The slow-rise, low-amplitude profiles produced by absorption-conjugation-recirculation are precisely the kind of inputs that favour redox signalling over wholesale oxidation. Small, repeated excursions can engage peroxiredoxin-based relays and the Keap1-NRF2 axis without collapsing cellular thiol buffers, while extensive conjugation restrains futile redox cycling [37].

These features also explain why chemical antioxidant assays overestimate what polyphenols will do *in vivo*: the species present in plasma are different molecules with different kinetics and targets. Recognizing conjugation not as a loss of function but as a regulatory layer helps reconcile the modest plasma levels seen after realistic intakes with durable, adaptive phenotypes in tissues [38].

### 4.2. Peripheral H_2_O_2_ Generation and Aquaporin Entry: Implications for ARE/NRF2 and PTPs

Outside the test tube, most of the oxidants that matter to signalling are made close to membranes or in the extracellular space. Polyphenols participate in that geography in two ways. First, catechol- and gallate-type structures can autoxidize at physiological pH, especially in metal-rich or highly oxygenated niches, yielding superoxide that dismutates to hydrogen peroxide. Second, polyphenols shape enzyme activities that already produce oxidants, xanthine oxidase, monoamine oxidase, and several NADPH oxidases, either by acting as substrates for peroxidase cycles or by modulating cofactor availability. The net effect is small, local pulses of H_2_O_2_ near cell surfaces and at organelle contact sites rather than a uniform bath of oxidants [39].

**Figure 7:**
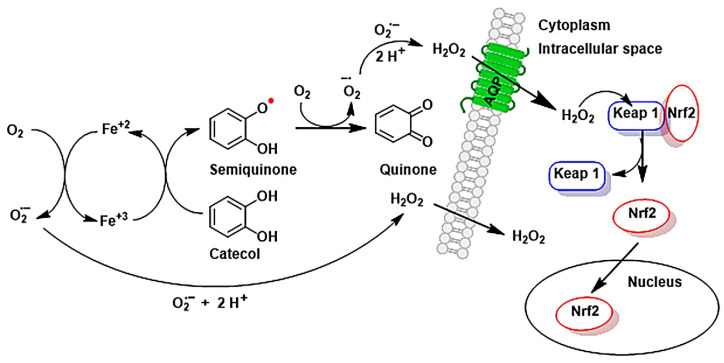
The oxidation of catechol to quinone generates the superoxide radical (O_2•_^−^) as a by-product. During metal ion-mediated oxidation, the oxidation of catechol to o-semiquinone generates O_2•_^−^, that can be converted to the less reactive H_2_O_2_ in the presence of protons or water. The right side of the figure shows a diagram describing the entry of H_2_O_2_ facilitated by aquaporin and NRF2 cell signalling.

Those pulses become biologically legible because H_2_O_2_ can cross membranes through “peroxiporins”, a subset of aquaporins (notably AQP3, AQP8, and AQP9). Passive diffusion through the bilayer is slow; peroxiporins provide a gated conduit that couples extracellular chemistry to intracellular targets within seconds. These three isoforms are not uniformly distributed across tissues. AQP3 is prominent in keratinocytes and other epithelial barriers, AQP8 is enriched in hepatocyte mitochondrial membranes and in leukocytes, and AQP9 appears mainly in liver and myeloid cells, so the tissue in which peroxiporin-facilitated signalling operates is not the same from one cell type to the next. Direct oxidation of a target cysteine by H_2_O_2_ is also comparatively rare on its own, because that reaction is intrinsically slow. Most physiological signalling instead proceeds through peroxiredoxins, which react with H_2_O_2_ far faster and then relay the oxidising equivalent onward through thiol exchange, a distinction worth keeping in mind when interpreting experiments that measure bulk H_2_O_2_ flux rather than the identity of the protein actually modified. Equally important are the sinks: peroxiredoxins and glutathione peroxidases intercept H_2_O_2_ immediately, keeping concentrations low and confining the signal to microdomains. What slips past, or is deliberately relayed by oxidized peroxiredoxins, selectively modifies redox-sensitive cysteines (Figure 7) [40].

Two families sit at the receiving end. Protein tyrosine phosphatases (PTPs) carry an active-site cysteine with an unusually low pKa, transient oxidation to sulphenic acid, and often, S-glutathionylation inactivates them until cellular reductants reverse the change. Brief PTP inhibition amplifies kinase cascades from growth factor and cytokine receptors, shifting phosphorylation set points without wholesale damage. In parallel, the Keap1-NRF2 system reads electrophilic and oxidative tone [41].

H_2_O_2_ itself is a weak direct modifier of Keap1, but redox relays and modest electrophile formation downstream of polyphenol oxidation nudge Keap1’s cysteines, slow NRF2 turnover, and expand the antioxidant transcriptome (NQO1, HO-1, glutathione GSH synthesis genes, cystine transport). The same logic that enables benefit also sets limits: if extracellular generation overwhelms peroxiporin-fed buffering, thiol pools are drained, futile redox cycling emerges, and signalling blurs into stress. In practice, physiological intakes produce short, low-amplitude H_2_O_2_ transients, exactly the regime in which peroxiporins translate peripheral chemistry into adaptive intracellular responses [42].

### 4.3. Phase II Enzymes and Quinone Fate: GST, COMT, UGTs, SULTs, Synthesis of NQO1’s Role

**Figure 8:**
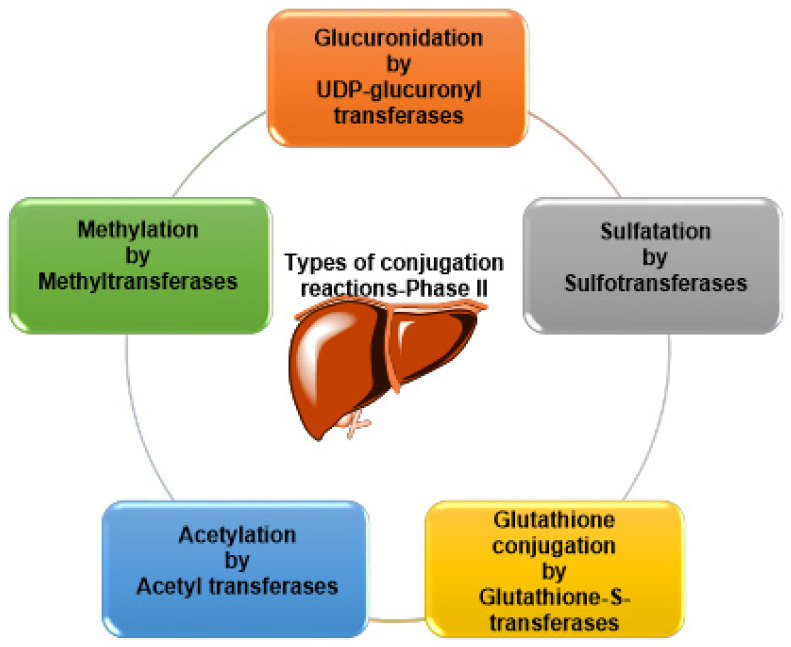
Phase II biotransformation reactions, also called conjugation reactions, in which glucuronidation and sulphation attach polar groups to phenolic hydroxyls, lowering reactivity and preparing the resulting conjugates for transport and excretion.

Once phenolic substrates edge toward quinone formation, the cell’s phase II machinery decides whether that chemistry is neutralized, repurposed, or allowed to escalate. The most protective path begins upstream of overt damage: NQO1 reduces quinones directly to hydroquinones through an obligate two-electron transfer. By skipping the semiquinone state, this reaction short-circuits redox cycling with oxygen, lowers electrophilicity, and buys time for conjugation and export. In practice, NQO1 functions as a gatekeeper. When its activity is sufficient, often after NRF2 induction, quinone flux is diverted into detoxification rather than into thiol depletion and protein adduction (Figure 8) [43].

Glucuronidation and sulphation provide the primary exit routes. UDP-glucuronosyltransferases (UGT1A/UGT2B families) append glucuronic acid to phenolic hydroxyls on aglycones and, after NQO1 repair, on hydroquinones. The resulting O-glucuronides are bulkier, more polar, and far less prone to autoxidation; they are also excellent substrates for MRP transporters that lower intracellular residence time. Sulphotransferases (chiefly SULT1A1 and related isoforms) perform an analogous edit using PAPS as sulphate donor [44].

Sulphates are typically formed at lower substrate concentrations than glucuronides and can dominate when catalytic capacity is not saturated. Both reactions remodel not only pharmacokinetics but also mechanism: by masking hydrogen-donating hydroxyls and fortifying polarity, they nudge polyphenols away from direct radical chemistry and toward regulated, compartmentalized signalling [45].

When electrophilic load rises, glutathione S-transferases become crucial. GSTs catalyse Michael addition of GSH to quinones and quinone methides, forming thioether adducts that are generally less reactive and are funnelled into the mercapturic acid pathway (γ-glutamyl cleavage, cysteinyl-glycine trimming, N-acetylation) on their way to excretion. This route is a double-edged sword: it neutralizes electrophiles but consumes GSH, and if quinone formation outpaces GST and NQO1 capacity, the cell enters a futile cycle of GSH drain and peroxide production. COMT provides a complementary, pre-emptive defence specific to catechols [46].

By O-methylating one hydroxyl with S-adenosylmethionine, COMT converts highly redox-active catechols into less autoxidation-prone guaiacyl derivatives, easing the burden on NQO1 and the thiol pool. Seen together, these enzymes define a choreography, NQO1 repairs and pauses the chemistry, UGTs/SULTs and transporters usher products out, GSTs trap what escapes, and COMT reduces the upstream propensity to form quinones. The balance among them determines whether quinone traffic ends in quiet clearance or tips into cytotoxicity [47].

### 4.4. From Aglycone to Conjugate: Changes in Nucleophilicity/Electrophilicity and Thiol Reactivity

The passage from aglycone to conjugate rewrites the electronic personality of a polyphenol. Free phenolic rings donate electron density through their hydroxyl groups. Deprotonation to the phenolate strengthens that donation, raises nucleophilicity at oxygen, and lowers the energetic cost of one-electron transfer. These features underlie both the ease with which catechols yield semiquinones and the readiness of hydroquinones to climb toward p-quinones [48].

Conjugation reverses much of this. O-glucuronidation replaces the labile O-H with an ether-like linkage to a carboxylate-bearing sugar. The result is a strongly polar, anionic handle that withdraws electron density from the ring, attenuates resonance donation, and curbs formation of the phenolate. In practical terms, the conjugate is less nucleophilic at oxygen, less able to chelate metals through adjacent oxygens, and markedly less prone to autoxidize [49].

Sulphation pushes in the same direction, often more decisively. A phenolic sulphate ester introduces a dianionic, hard, electron-withdrawing group that depresses the basicity of the aromatic oxygen and further disfavours radical chemistry. Because both glucuronides and sulphates mask the very sites that participate in redox cycling, the route to electrophilic quinones is largely blocked unless those groups are first removed. Where peroxidases or redox-active metals could coax an aglycone toward a quinone, the conjugate tends to sit out the reaction [50].

Thiol reactivity tracks these changes. Aglycone-derived o- or p-quinones are “soft” electrophiles that react efficiently with the soft nucleophile of cysteine, yielding Michael adducts on glutathione and on protein thiols that tune, or, at high load, derail, function. Conjugation largely prevents quinone formation and thus severs this line to covalent thiol chemistry. There are two caveats. First, β-glucuronidases and sulphatases can regenerate aglycones in acidic or inflamed microenvironments, restoring local electrophile potential without raising systemic exposure [51].

Second, mixed speciation *in vivo* means conjugates coexist with small pools of free aglycone; even modest deconjugation at membranes can produce microdomain-level pulses of electrophile capable of nudging Keap1 or transiently glutathionylating redox-sensitive proteins. The net effect is a shift from direct, diffuse thiol targeting toward context-dependent, spatially gated interactions, with conjugation acting less as a terminus than as a reversible safety catch [52].

## 5. Polyphenols and Microbiota: Metabotypes and Phenolic Metabolites

Before we discuss specific pathways, we need to reframe the gut as a chemical reactor that finishes what the host cannot. Most dietary polyphenols reach the colon at least partly intact, still glycosylated, esterified, or embedded in cell-wall material, and meet consortia that supply the missing functions: glycosidases to unmask aglycones, ring-cleaving enzymes, reductases, and dehydroxylases. The products of this relay are not miniature versions of the parents but new molecules with distinct permeability and targets, many of them less prone to autoxidation and better suited to circulate at low micromolar levels for hours [53].

Crucially, the capacity to make these “signalling metabolites” is uneven across people. Stable metabotypes determine whether urolithins, phenyl-γ-valerolactones, or simpler phenyl-acids dominate after a given meal. That variability is not noise but a determinant of biology, setting exposure profiles that shape redox tone, NRF2 readiness, and ultimately which clinical outcomes are even possible [54].

### 5.1. From Food to Colon: Microbial Transformation and “Signalling Metabolites”

Most dietary polyphenols are not built to slip quickly through the small intestine. They arrive embedded in plant cell walls, bound to sugars or larger tannin scaffolds, and often travel with fibre that slows access to digestive enzymes. As a result, a substantial fraction escapes early absorption and proceeds to the colon, where the metabolic centre of gravity shifts from host to microbes. There, specialized consortia hydrolyse glycosides, open heterocyclic rings, and perform reductive and dehydroxylating steps that human enzymes rarely manage [55].

**Figure 9:**
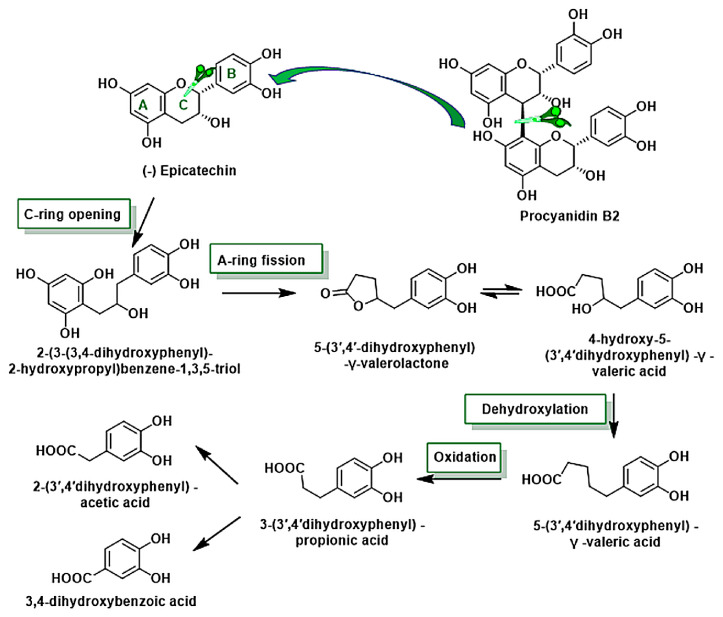
Metabolic pathways of intestinal microbial metabolism of epicatechin, monomeric flavon-3-ols and procyanidin units. The diagram represents the study of colonic degradation of epicatechin and urinary excretion of catabolites in humans. The predominant degradation products were (-)-5-(3′,4′-dihydroxyphenyl)-γ-valerolactone, 5-(3,4-dihydroxyphenyl)-γ-valeric acid, 3-(3-hydroxyphenyl)propionic acid, and derivatives of phenylacetic acid and benzoic acid.

Ellagitannins and ellagic acid, for instance, are converted through successive dehydroxylations and lactone rearrangements into the urolithins, small dibenzopyran-6-one derivatives that cross the epithelium readily. Flavan-3-ols and procyanidins undergo C-ring cleavage to yield phenyl-γ-valerolactones and, downstream, a family of phenylacetic and phenylpropionic acids. Even complex flavonols eventually converge on simpler phenyl-acids, some of which persist in plasma far longer than their precursors (Figure 9) [56].

These transformations matter because they recast bioactivity. Microbial metabolites are less prone to autoxidation and metal redox cycling than their parent aglycones. Instead, they behave as relatively stable, low-micromolar messengers that interact with cellular targets [57].

Urolithin A is emblematic: beyond modest antioxidant tone, it engages stress-response circuitry and mitochondrial quality-control pathways associated with improved cellular housekeeping [58].

Phenyl-γ-valerolactones and phenyl-acids, for their part, modulate transcriptional programs linked to redox and inflammatory balance and can influence endothelial and metabolic signalling in ways not predictable from the starting flavanols [59].

Crucially, production of these metabolites varies widely between individuals, a pattern of “metabotypes” that reflects the presence or absence of key bacterial guilds. The same food can thus seed distinct systemic profiles, with consequences for efficacy in trials and for day-to-day responses. Seeing the colon not as a sink but as a bioreactor reframes polyphenols from direct radical scavengers to precursors of signalling molecules. It also provides a practical handle for precision nutrition: the phenotype that reaches tissues depends less on the label of the phytochemical and more on who is there to finish the chemistry [60].

### 5.2. Metabotypes and Interindividual Variability

“Metabotype” here refers to a reproducible, person-specific pattern of microbial biotransformation that governs which phenolic metabolites appear after a given intake and in what amounts. The idea is easiest to grasp with ellagitannins: some individuals consistently generate urolithin A as the dominant product, others form broader mixtures that include urolithin B and isourolithins, and a third group produces little to none. Analogous partitions exist for flavan-3-ols, where conversion to phenyl-γ-valerolactones and downstream phenyl-acids varies widely across people [61].

These phenotypes are not mere curiosities; they shape exposure profiles, peak concentrations, time to peak, and area under the curve, and, by extension, the probability that signalling thresholds for cellular responses are reached in target tissues [62].

Several layers of biology create and maintain metabotypes. Microbial membership is central, but function depends on networks: glycosidases to unmask aglycones, ring-cleaving enzymes, dehydroxylases, and reductases that act in sequence. Diet supplies both substrates and ecological pressure, with fibre and co-ingested macronutrients altering transit time, pH, and redox tone in the colon [63].

Medications, especially antibiotics and proton-pump inhibitors, can reset communities for weeks to months. Host factors add another filter. Polymorphisms in UGT, SULT, and COMT isoforms influence the fate of absorbed metabolites, transporter expression at the gut-liver axis modulates recirculation, and mucosal β-glucuronidase and sulphatase activities shape the balance between conjugated and free species at the epithelial surface. The result is a coupled host-microbe system in which the same food dose yields distinct molecular fingerprints in plasma and urine [64].

From a translational standpoint, metabotypes explain why large nutrition trials often report modest average effects with large variance. Non-producers are not necessarily non-responders, but their pathways to benefit may depend more on alternative metabolites or on indirect redox signalling from low-level peroxide flux, rather than on a flagship compound [65].

Two practical remedies follow. First, stratifying participants by baseline metabolite production, or even by simple post-dose metabolite readouts, can unmask effects otherwise diluted by heterogeneity. Second, interventions that shift function (prebiotics, fermented carriers, longer run-ins) may convert “silent” metabotypes into producers, moving individuals into the exposure window where redox-signalling programs are most reliably engaged [66].

### 5.3. Relevance to the Pro-/Antioxidant Axis and NRF2 Activation

Microbial conversion reshapes not only what circulates after a polyphenol-rich meal but also how the organism sits on the pro-/antioxidant axis. Aglycones and catechol-rich precursors are chemically poised to autoxidize, generate semiquinones, and leak hydrogen peroxide, useful at low amplitude for signalling, hazardous when metals are abundant or thiols are scarce [35].

By contrast, dominant colonic products such as urolithin A and families of phenyl-γ-valerolactones and phenyl-acids are comparatively stable: they chelate poorly, autoxidize reluctantly, and reach tissues as low-micromolar, long-tail exposures. That shift dampens the propensity for futile redox cycling and tilts the system away from diffuse oxidative pressure toward regulated, pathway-level responses [67].

NRF2 sits at the centre of that recalibration, and two inputs matter. First, modest extracellular H_2_O_2_ transients, arising from residual autoxidation or from enzyme systems that polyphenols modulate, still traverse peroxiporin channels and engage redox relays, nudging the Keap1 sensor without collapsing the glutathione buffer. Second, certain microbial metabolites carry their own “gentle push” [68].

**Figure 10:**
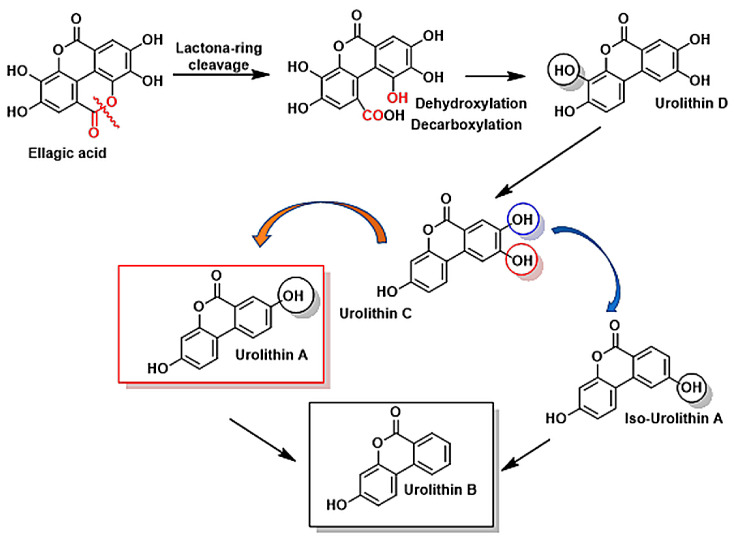
Formation of urolithins from ellagic acid in the intestine. After ingesting foods containing ellagitannins, these are hydrolysed in the stomach to produce ellagic acid, which undergoes a series of transformations by the intestinal microbiota to form different urolithins.

Urolithin A is the clearest case: it promotes mitochondrial quality control and lowers basal oxidative tone, a context that tends to favour adaptive NRF2 signalling over injury responses (Figure 10) [69].

Phenyl-γ-valerolactones and downstream phenyl-acids also influence inflammatory and endothelial set points, which indirectly tunes the balance between oxidant production and detoxification gene expression. In practice, these species act less as radical sponges than as tone-setters, helping cells maintain a state in which NRF2 target genes, NQO1, HO-1, GCL subunits, cystine transport, are easier to mobilize and quicker to resolve [70].

Metabotypes then determine who reaches that sweet spot at realistic intakes. Individuals who efficiently generate urolithins or sustained phenyl-acid profiles are more likely to cross signalling thresholds for NRF2 without incurring electrophile burden, whereas non-producers may rely on sporadic deconjugation of host-formed glucuronides at inflamed interfaces to achieve local Keap1 modification [71].

Both routes can work, but they differ in collateral costs: the microbial path is frugal with thiols and NAD(P)H, the deconjugation path risks local adduct pressure. Recognizing these alternatives explains the heterogeneity of human responses and points to an actionable principle: in the polyphenol field, “antioxidant” benefit often arrives not by quenching oxidants, but by steering cellular programs, NRF2 foremost among them, into readiness with minimal oxidative spend [72].

## 6. Quinone Forms, Adducts, and Safety

Before diving into particulars, it is worth clarifying what we mean by “quinone forms” and why they sit at the fulcrum of benefit and risk. Oxidation of catechol and hydroquinone motifs yields o- and p-quinones, electron-poor, conjugated carbonyl systems that behave as soft electrophiles toward thiolate nucleophiles. In living cells, this chemistry is not free-running: NQO1 can return quinones to their reduced partners via two-electron repair, glutathione intercepts electrophiles to form exportable thioethers, and conjugation plus transport lowers residence time [24].

Whether quinone traffic remains a useful nudge to redox signalling or drifts into injury depends on rates and context, how quickly quinones are formed, how abundant and accessible protein cysteines are, the state of the glutathione/NADPH economy, and the presence of redox-active metals that favour one-electron detours. Adducts themselves are ambiguous: at low flux, they can act as reversible edits that bias sensors like Keap1, whereas sustained pressure hardens into enzyme inhibition, crosslinking, proteostasis strain, and, in the nucleus, lesions that complicate repair. With that frame, we now examine o-quinones, the most proximate electrophiles in many polyphenol systems, their preferences for GSH and protein cysteines, and the functional consequences that follow [73].

### 6.1. o-Quinones: Electrophilicity, Adducts with GSH and Protein Cysteines, Functional Consequences

**Figure 11:**
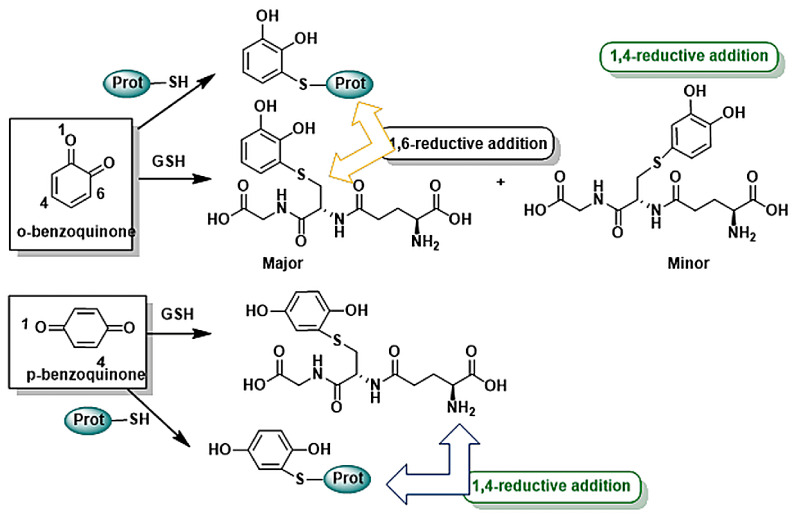
Quinones react with cellular nucleophiles (GSH, protein sulphydryls). The diagram shows the thia-Michael addition of L-cysteine to quinones and of GSH to quinones. Unsubstituted o-quinones undergo 1,6-reductive addition reactions with nucleophilic thiols due to their prolonged conjugation. However, 1,4-reductive addition is often observed as a minor product in these reactions. p-Quinones typically undergo 1,4-reductive addition reactions, which regenerate hydroquinone through the formation of a covalent bond with the cysteine residue.

o-Quinones are prototypical “soft” electrophiles. By conjugating two carbonyl groups to an aromatic ring, they distribute positive character across the β-carbons and act as Michael acceptors that favour reaction with soft nucleophiles, chiefly thiolates. In aqueous buffers near neutrality, the reaction landscape is set by speciation on both sides: phenolic precursors that form o-quinones more readily at higher pH, and cysteines whose local environment depresses pKa to generate reactive thiolates at physiological pH (Figure 11) [74].

The result is swift S-quinonylation at accessible protein cysteines, in kinetic competition with glutathione (GSH). Because cellular GSH resides at millimolar levels, it typically captures a substantial share of quinone flux, forming GS-adducts that enter the mercapturic-acid pathway; however, low-pKa cysteines in active sites or protein-protein interfaces can rival GSH locally and become privileged targets [75].

Adduct formation is not merely a scavenging event, it edits function. On Keap1, S-quinonylation at sensor cysteines weakens the E3 ligase assembly and stabilizes NRF2, shifting cells toward an adaptive transcriptome (NQO1, HO-1, GSH synthesis, cystine transport). Elsewhere, the same chemistry can be disruptive [76].

Covalent modification of catalytic cysteines in enzymes such as GAPDH, protein tyrosine phosphatases, or mitochondrial dehydrogenases blunts flux through glycolytic and redox circuits. With sustained exposure, secondary reactions, oxidation of thioether adducts, crosslinking to lysine or additional cysteines, and polymerization, yield “quinoproteins” that resist repair and burden the proteostasis systems. Depletion of GSH compounds the problem, lowering the threshold for peroxide damage and impairing disulphide repair machineries [77].

Reversibility is context-dependent. Some S-quinonylations undergo retro-Michael reactions in a thiol-rich milieu or are diluted by turnover and proteasomal clearance; others are effectively permanent on biological timescales. Reductive pressure from NAD(P)H and NQO1 can short-circuit adduct accrual by returning quinones to hydroquinones, while GSTs accelerate GSH capture and export [78].

The balance among these sinks determines whether o-quinone formation functions as a useful electrophilic “nudge” that biases NRF2 and detoxication, or drifts into cytotoxicity marked by thiol drain, enzyme inactivation, and protein crosslinking. In practice, brief, low-dose pulses are tractable and adaptive, persistent generation in metal-rich or thiol-poor settings is not [79].

### 6.2. Potential Genotoxicity: ROS, DNA Breaks, Adducts, and Nuclear Translocation of Specific o-Quinones

Genotoxic risk from polyphenol oxidation arises along two converging lines: indirect damage driven by reactive oxygen species (ROS) and direct covalent chemistry of the quinone itself. On the ROS side, catechols that redox cycle through semiquinones reduce O_2_ to superoxide and, by dismutation, to H_2_O_2_. In the presence of labile iron or copper, H_2_O_2_ feeds Fenton chemistry to generate hydroxyl equivalents that attack the deoxyribose backbone and oxidize nucleobases [80].

**Figure 12:**
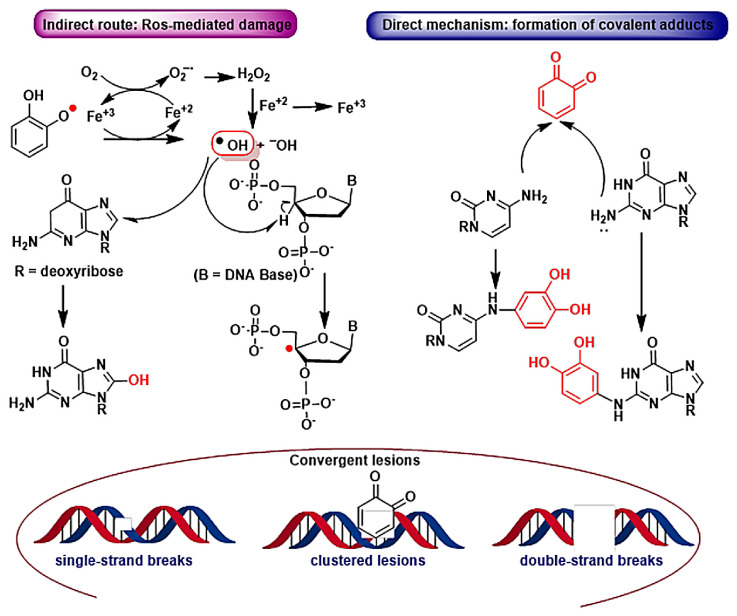
Quinones derived from polyphenols cause DNA damage through two principal pathways, namely direct electrophilic attack and indirect oxidative stress. The indirect mechanism is driven by the instability of semiquinones and their interaction with molecular oxygen. Semiquinones are formed from polyphenol-derived quinones, which are reduced by cellular enzymes or reducing agents to generate semiquinone radicals. These semiquinones rapidly transfer single electrons to molecular oxygen, generating superoxide anions. These anions undergo spontaneous dismutation, or dismutation catalysed by superoxide dismutase, to form hydrogen peroxide, and the hydrogen peroxide produced then fuels cellular Fenton chemistry, which depends on intracellular transition metals such as iron or copper. The Fenton reaction drives the conversion of hydrogen peroxide into highly reactive hydroxyl radicals, which attack and oxidise the DNA molecule. This process leads to strand breaks, modifications to the sugars, such as oxidation of the deoxyribose backbone, and alterations to the nitrogenous bases, for example, the formation of 8-oxo-7,8-dihydroguanine. The direct pathway operates through structural reactivity, since many quinones act as potent Michael acceptors. They can bind directly and covalently to nucleophilic sites on the DNA bases, namely purines and pyrimidines, and this direct conjugation gives rise to bulky DNA adducts that distort the DNA double helix, cause replication errors, and can lead to mutations if they are not repaired by cellular mechanisms.

Figure 12 now depicts the two converging routes described above, showing how semiquinone-driven superoxide and hydrogen peroxide feed Fenton chemistry to oxidise the deoxyribose backbone and nucleobases on one side, while direct quinone adduction at N7 of guanine and N3 of adenine promotes depurination and abasic site formation on the other. Both routes are shown converging on the same downstream outcomes, namely single-strand breaks, clustered lesions, and double-strand breaks that recruit ATM/ATR signalling.

The immediate lesions include single-strand breaks, abasic sites, and 8-oxo-7,8-dihydroguanine; clustered damage or coincident nicks on opposing strands escalate to double-strand breaks that recruit ATM/ATR signalling, γH2AX formation, and checkpoint activation. If the oxidative burst is transient, base-excision and single-strand break repair can keep pace; if it is sustained, repair enzymes themselves become collateral targets as thiol oxidation and NAD(P)H drain slow the system [81].

Quinones add a second layer through electrophilic attack. o-Quinones are soft Michael acceptors that prefer thiolates, but at sufficiently high local concentrations or in thiol-depleted niches, they can react with nucleobases, particularly at N7 of guanine and N3 of adenine, forming adducts that destabilize the glycosidic bond and promote depurination. The resulting abasic sites are intrinsically mutagenic during replication [82].

It is worth noting that this precise adduction chemistry at N7 of guanine and N3 of adenine has been characterised in most detail for endogenous catechol oestrogen quinones rather than for dietary polyphenol-derived quinones directly. We therefore present it here as a plausible chemical analogy grounded in shared quinone reactivity, rather than as evidence obtained from dietary polyphenols themselves, and direct confirmation in polyphenol-derived quinones remains an open question for future work.

Quinone-protein crosslinks in chromatin further complicate repair by stiffening local structure and sequestering factors needed for lesion processing. Topoisomerase poisoning has been reported for some flavonoid-derived species, generating cleavage complexes that convert into double-strand breaks upon collision with replication forks, a mechanistic detour that does not require high ROS [83].

This topoisomerase-poisoning mechanism was likewise demonstrated using synthetic electrophilic alkaloids rather than dietary polyphenols, so we present it as an illustration of what quinone electrophiles can do in principle. Direct evidence that dietary polyphenol-derived quinones poison topoisomerases through this same route is still lacking and would need dedicated study.

Whether these events reach biological consequence depends on access to the nucleus and on intracellular countermeasures. Small, relatively lipophilic quinones can diffuse across nuclear pores; others arrive effectively “piggy-backed” as adducts on proteins that shuttle to chromatin [84].

**Figure 13:**
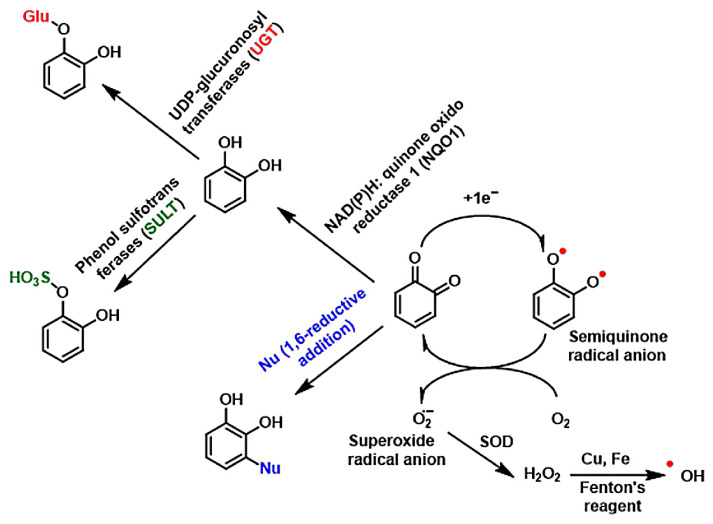
Mechanisms of quinone toxicity, showing how excessive quinone formation depletes glutathione, damages proteins through covalent adduction, and drives redox cycling that generates further reactive oxygen species within the cell.

In many cells, however, NQO1 suppresses the problem by funnelling quinones into two-electron reduction, and UGT/SULT pathways lower re-entry by converting the pool into conjugates that poorly penetrate the nucleus. Context therefore rules: genotoxicity is most likely when quinone formation outstrips NQO1 and conjugation capacity, when redox-active metals are abundant, or when local deconjugation regenerates aglycones in inflamed or hypoxic microenvironments (Figure 13) [85].

### 6.3. The “Safety Window” and Conditions That Exacerbate Risk (Metals, Hypoxia, GSH Depletion)

We present the safety window that follows as a conceptual heuristic rather than as a set of validated numeric thresholds, since compound-specific concentrations, exposure durations, and tissue-level biomarkers that would fix its boundaries precisely have not yet been established across the polyphenol classes discussed in this review. A biomarker-anchored version of this framework would need to specify circulating conjugate concentrations, the glutathione to glutathione disulphide ratio, and markers of oxidative DNA damage such as 8-oxo-7,8-dihydro-2’-deoxyguanosine, measured alongside the contextual factors described below. We flag this as a priority for future validation rather than a claim already supported by such measurements.

The same chemistry that lets polyphenols prime defences also sets the terms of their safe use. A practical way to think about this is as a “safety window”, bounded at the low end by doses too small to move redox programs and at the high end by conditions that convert gentle signalling into injurious chemistry. Within that window, transient peroxide pulses and limited electrophile formation nudge Keap1-NRF2, refill glutathione, and upregulate repair without exhausting reducing power. Outside it, the system tips: quinone formation outpaces two-electron repair, futile redox cycling drains NAD(P)H, and covalent pressure falls on protein thiols and DNA [86].

Three contexts narrow the window most predictably. Redox-active metals are the first. Labile iron and copper accelerate one-electron pathways, turning semiquinones into efficient O_2_ reducers and feeding Fenton chemistry that propagates strand breaks and lipid peroxidation. Notably, metal speciation matters more than total body stores: haemolysis, tissue injury, and inflamed plaques create microdomains rich in loosely liganded Fe^2+^/Cu^+^ where otherwise tolerable exposures become problematic. Tight chelation by proteins keeps risk low, while displacement by phenolates at higher pH can do the opposite [87].

Hypoxia is the second. Although less oxygen might seem protective, hypoxic niches often carry reductive stress, acidosis, and bursts of β-glucuronidase from immune cells. The combination regenerates aglycones from circulating conjugates at the very interface where oxygen re-entry is intermittent and iron is labile-fertile ground for quinone formation and inefficient re-oxidation. Hypoxia also compromises mitochondrial and peroxisomal peroxide clearance and can depress NADPH supply, weakening NQO1-mediated “two-electron refuge” [88].

Depletion of glutathione is the third. When GSH is low, whether from diet, disease, drug interactions, or sustained inflammatory tone, two buffers vanish at once: the capacity to intercept quinones as benign thioethers and the ability to restore oxidized cysteines on enzymes and signalling proteins. Under these constraints, even modest electrophile traffic translates into enzyme inhibition, proteostasis strain, and impaired DNA repair [89].

The practical implication is straightforward. Benefit depends as much on context as on dose: adequate thiol and NADPH reserves, restrained metal availability, and intact conjugation/transport systems widen the safety window, whereas metal-rich, hypoxic, or GSH-poor settings shrink it and magnify risk. Careful attention to these backgrounds helps explain discordant findings and guides where polyphenol strategies are likely to help, or to harm [90].

## 7. Oxidative Enzymes in Foods and Processing

Before considering human biology, we need to acknowledge that a substantial portion of “polyphenol chemistry” happens before ingestion, during harvesting, storage, and processing. Plant oxidative enzymes, chiefly polyphenol oxidase (PPO), but also peroxidases and, in some fermentations, fungal laccases, govern whether native diphenols persist as reversible redox couples or are driven into short-lived quinones that rapidly adduct to proteins and polymerize into brown pigments [91].

This enzymic layer is exquisitely sensitive to variables that processors can control: tissue disruption (which collapses compartmentation), dissolved oxygen, pH, temperature-time profiles, water activity, and trace Fe/Cu that catalyse one-electron detours. The outcome is not merely cosmetic. Quinone speciation influences texture, aroma retention, and astringency, and it sets the fraction of phenolics that remains biologically tractable after digestion [92].

Because standard antioxidant assays reward electron transfer regardless of provenance, they can rise even as the pool of recyclable diphenols shrinks. With that in mind, we begin with PPO, the dominant catalyst of enzymatic browning in fresh and minimally processed matrices, its mechanism, kinetics, and the nutritional and sensory consequences of its activity [93].

### 7.1. PPO and Browning: Mechanisms, Kinetics, and Nutritional/Organoleptic Quality

Polyphenol oxidase (PPO) is the engine that turns tissue disruption into visible browning. It is a dicopper oxidase embedded in plastids that catalyses two related reactions: the monophenolase (cresolase) activity that ortho-hydroxylates monophenols to o-diphenols, and the diphenolase (catechol oxidase) activity that oxidizes o-diphenols to o-quinones using molecular oxygen as co-substrate. Those quinones are highly electrophilic; they undergo rapid cyclizations and Michael additions with amino and thiol groups on proteins, and they polymerize to brown, melanin-like pigments. Because the reactive pool forms at the very surface where cells are cut or bruised, a small enzymatic flux can have outsized sensory effects [94].

The kinetics have a few practical signatures. First, many substrates show a lag phase that reflects the need to generate the first o-diphenol before diphenolase turnover dominates; pre-existing o-diphenols shorten that delay [95].

Second, oxygen tension and pH tune both rate and speciation: activity typically climbs from acidic toward near-neutral pH and increases with dissolved O_2_, but rises in pH also favour quinone persistence and protein adduction [96].

**Figure 14:**
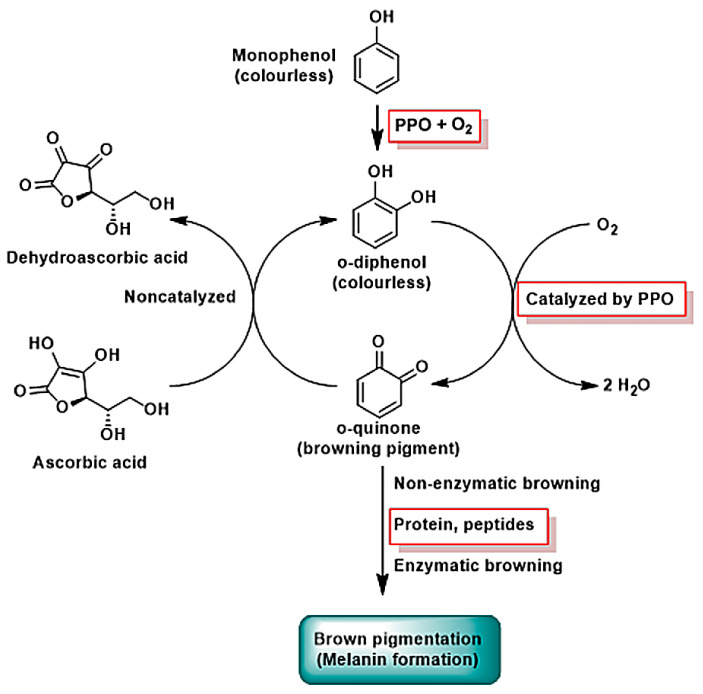
Diagram of the enzymatic browning process and reduction of o-quinone to o-diphenol by ascorbic acid, illustrating how this reaction protects food quality by limiting the accumulation of reactive quinone species during processing.

Third, the enzyme is “self-limiting” at high product load: o-quinones can inactivate PPO by modifying active-site residues, while ascorbate recycles quinones back to diphenols, altering apparent V_max_ and masking true catalytic capacity. Temperature and tissue microstructure matter as well; blanching collapses activity by denaturation, whereas intact cell barriers slow substrate-enzyme encounter (Figure 14) [97].

Browning is not merely cosmetic. Quinone-protein reactions consume lysine and cysteine side chains, stiffen texture, and bind aroma compounds, lowering perceived freshness. Nutritionally, PPO-driven chemistry depletes native polyphenols and can reduce their bioavailability by forming insoluble complexes with proteins and cell-wall polymers. Yet the story is nuanced: controlled oxidation may temper astringency in some matrices and can stabilize colour once polymers are formed. From a health perspective, PPO shifts the phenolic pool toward quinone and adduct species, changing both antioxidant readouts and the likely signalling footprint downstream [98].

### 7.2. Processing Steps That Shift Quinone Speciation and Net Antioxidant Capacity

Processing determines whether polyphenols drift toward transient o-quinones that can be recycled, or toward long-lived adducts and pigments that are effectively terminal. The first inflection point is physical disruption. Cutting, crushing, and juicing collapse compartmentation, mix substrates with polyphenol oxidase (PPO) and trace metals, and flood the system with oxygen. Within minutes, o-quinones accumulate and begin to partition: some are reduced back by endogenous ascorbate, others add to cysteinyl/lysyl residues on proteins or polymerize into melanin-like pigments [74].

The balance can be shifted by temperature. Brief blanching denatures PPO and preserves native phenolics, whereas prolonged heating accelerates non-enzymatic autoxidation and fosters crosslinking. Heat also softens cell walls and increases extractability, so measured antioxidant capacity may rise despite chemical losses, an artefact of greater solubility and assay access rather than of a larger pool of intact, bioavailable molecules [99].

pH and oxygen control are the next levers. Acidification slows PPO and suppresses phenolate formation, curbing autoxidation and quinone persistence; lowering headspace O_2_ (vacuum or modified atmospheres) has similar effects. Chelators such as citrate or EDTA do not strip PPO’s catalytic copper but do sequester free Fe/Cu that catalyse one-electron routes, reducing futile redox cycling. Reducing agents and nucleophiles change speciation more directly: ascorbate returns quinones to diphenols but can dominate electron-transfer assays; sulphites, where permitted, form sulphonated adducts that “bleach” quinones at the cost of erasing electrophilic signalling [100].

Modern “minimal” processes are not neutral. High-pressure processing often preserves a fresh matrix but can leave PPO partly active, so quinone formation resumes during storage unless combined with mild heat or acid. Fermentation can go either way: microbial glycosidases and esterases liberate phenolics and generate new, often more stable metabolites, while fungal laccases or plant peroxidases drive deeper oxidation into high-molecular-weight pigments. Drying and roasting shut down enzymes yet favour non-enzymatic coupling and Maillard products that contribute to radical-scavenging readouts but typically lower phenolic bioavailability [101].

In practice, “net antioxidant capacity” is the moving sum of opposing changes, enzyme inactivation, extractability, quinone recycling, and irreversible adduction. Because standard assays reward electron transfer regardless of provenance, they can rise when speciation has shifted away from reversible, biologically tractable pools. Interpreting gains or losses therefore requires asking not only “how much activity”, but “which species now carry it, and can they still act usefully *in vivo*?” [102].

### 7.3. Implications for Polyphenol-Rich Matrices (Comparative Cases)

Matrix composition and microstructure largely determine whether polyphenols persist as reversible redox couples or are driven toward quinone adducts and inert pigments. In fresh pome fruits such as apple and pear, high PPO activity, abundant oxygen at cut surfaces, and plentiful protein targets conspire to accelerate o-quinone formation and browning after minimal disruption [103].

Clarification and filtration of juices can lower protein and particulate nucleophiles, tempering adduct formation, whereas mild acidification preserves diphenols by slowing both PPO turnover and non-enzymatic autoxidation. Berries sit at a different corner of the landscape [104].

Their lower pH, dense pectin network, and anthocyanin co-pigments alter speciation and diffusion: PPO acts more slowly, polymeric pigments form with different chromophores, and pasteurization that inactivates enzymes often increases measured antioxidant capacity by improving extractability even as it reduces the pool of native monomers. The net effect is that fruit purées and concentrates may score higher in electron-transfer assays while carrying fewer biologically tractable catechols [105].

Tea and cocoa illustrate how process choice deliberately steers chemistry. Green tea arrests PPO early (steaming or pan-firing), conserving catechins that readily enter host conjugation pathways. Black tea invites enzymatic oxidation during withering and rolling, building theaflavins and thearubigins via quinone coupling [106].

These larger products colour infusions and perform well in chemical assays yet behave differently *in vivo*, leaning more on colonic metabolism to phenyl-γ-valerolactones and phenyl-acids. Cocoa follows a comparable arc: fermentation and roasting reshape flavanol profiles, introduce Maillard partners, and foster high-molecular-weight pigments that alter astringency and protein interactions. Co-ingestion with dairy or other protein-rich carriers can further sequester reactive phenolics, lowering immediate browning and astringency while shifting absorption toward the distal gut [107].

Olive products highlight how a lipid matrix modulates redox. In extra-virgin olive oil, phenolics such as hydroxytyrosol and its derivatives reside in a dispersed polar microphase with limited oxygen access; autoxidation is slower, and quinone traffic is curtailed until emulsification during digestion exposes them to aqueous, metal-bearing environments [108].

In fermented table olives, microbial turnover and brining move the balance toward smaller, more mobile phenolics that survive storage yet face renewed oxidation when the matrix is disrupted. Whole grains and pulses add a final contrast: a large fraction of phenolics is cross-linked to cell-wall polymers or proteins [109].

Thermal and enzymatic treatments liberate bound forms and raise assay readouts, but the same steps can promote irreversible coupling. Across these cases, “improvement” in chemical capacity does not guarantee a larger pool of reversible, signal-competent species. What matters is how processing and matrix together set the stage for either recyclable diphenols or terminal adducts [110].

## 8. Human Evidence 2015–2025

Before turning to individual trials, we set the stage for how human evidence should be read. “Exposure” in this context is not a food name or label claim but the verified appearance of circulating conjugates or microbial metabolites. Without such biomarkers, dose and adherence blur, and mechanisms are easy to misattribute. Trial design also has to respect timescales: endothelial and inflammatory set-points may respond within weeks, while muscle function and cognitive trajectories need months to shift. Matrix and co-ingestion influence proximal absorption versus colonic transformation, and interindividual metabotypes determine who ever reaches a signalling threshold at realistic intakes [111].

Endpoints matter: composite cardiovascular outcomes dilute pathway-specific effects, whereas validated physiological readouts, flow-mediated dilation, hsCRP, thiol redox, or NRF2 target expression, are closer to mechanism and often precede “hard” events. With those guardrails in mind, we first review randomized interventions that test cocoa flavanols and urolithin A/analogs as exemplars of mechanistic plausibility meeting clinical pragmatism [112].

### 8.1. Key Clinical Trials (e.g., Cocoa Flavanols, Urolithin A, Anthocyanins, Catechins, Quercetin, and Resveratrol and Analog Interventions)

Two programs illustrate how “polyphenol stories” are now being tested at clinical scale: cocoa flavanols for vascular outcomes and urolithin A for muscle and mitochondrial health. In the COcoa Supplement and Multivitamin Outcomes Study (COSMOS), 21,442 older adults were randomized to a cocoa extract delivering 500 mg/day total flavanols (including 80 mg (-)-epicatechin) or placebo for a median 3.6 years. The trial’s pre-specified primary endpoint, the composite of total cardiovascular events, was not significantly reduced, but a secondary endpoint, cardiovascular mortality, fell by 27% (HR 0.73, 95% CI 0.54-0.98). A per-protocol sensitivity analysis, which censors for nonadherence and therefore carries less weight than the intention-to-treat result, supported a reduction in the total events composite, with no safety signals [112].

These results align with smaller physiologic trials yet also impose welcome discipline on expectations: clinically meaningful benefits may show up first in “harder” endpoints like CV death rather than broad composites. An exploratory ancillary analysis, not part of the pre-specified hierarchy, now adds biologic plausibility, over 2 years, cocoa extract lowered high-sensitivity C-reactive protein by roughly 8% per year versus placebo, suggesting attenuation of low-grade inflammation that could contribute to the mortality signal. By contrast, COSMOS-Mind, a separate ancillary trial with its own pre-specified primary cognitive endpoint, found no cognitive advantage for cocoa extract, while a parallel multivitamin arm did improve global cognition, a useful reminder that not all endpoints track together [113].

Urolithin A (UA), the microbiome-derived ellagitannin metabolite and mitophagy activator, has advanced through two randomized trials that, while modest in size, are consistent in direction. In older adults (*n* = 66), 4 months of UA (1000 mg/day) improved skeletal muscle endurance and favourably shifted biomarkers of mitochondrial health and inflammation, all secondary or exploratory measures, whereas primary outcomes such as the six-minute walk and hand-muscle ATP production did not separate from placebo, underscoring that performance measures with multiple physiological determinants may be less sensitive over short intervals [114].

A companion trial in middle-aged adults reported ~12% gains in muscle strength and improved exercise performance together with biochemical evidence of enhanced mitochondrial quality, with good tolerability across studies. Together, these data support UA as a mechanistically coherent, safe intervention that nudges muscle toward better energetic resilience, warranting longer and more diverse trials to clarify who benefits most and which endpoints are most responsive [115].

Beyond cocoa flavanols and urolithin A, four additional polyphenol classes have reached randomised human trials with informative, and at times contrasting, results. Anthocyanins offer one of the clearer cardiometabolic signals available. In a six-month, double-blind, randomised controlled trial in adults with metabolic syndrome, daily consumption of an anthocyanin-rich blueberry intervention improved several biomarkers of cardiometabolic function relative to placebo, an effect that tracked with detectable circulating anthocyanin metabolites rather than with the nominal dose alone [116]. The size of the effect remained modest, and the trial again illustrates a recurring theme of this review, namely that measurable exposure predicts a physiological response more reliably than the amount ingested.

Catechins, tested mainly as green tea extract or as the purified compound epigallocatechin gallate, provide a useful lesson in how primary and secondary endpoints can diverge. In a placebo-controlled, randomised trial of 97 men with high-grade prostatic intraepithelial neoplasia, 12 months of a standardised catechin mixture delivering 400 mg EGCG per day did not reduce the primary composite rate of prostate cancer, yet a secondary composite endpoint restricted to men with high-grade lesions alone showed a significant reduction, alongside a favourable safety profile throughout the year of supplementation [117]. A null primary endpoint accompanied by a positive signal on a narrower, mechanistically motivated secondary endpoint recurs across several polyphenol classes discussed in this review, and argues for pre-specifying subgroup and mechanistic endpoints rather than relying on broad composites alone.

Quercetin has the most consistent trial evidence for blood pressure among the flavonols studied to date. A systematic review and meta-analysis of seven randomised, placebo-controlled trials involving nearly 600 participants found significant reductions in both systolic and diastolic blood pressure, with the effect appearing somewhat greater at daily doses above 500 mg, although the authors cautioned that the clinical relevance of these reductions and the optimal treatment duration remain to be established [118].

Resveratrol illustrates something close to the opposite pattern, namely early promise followed by null results once larger and longer trials were conducted. Several smaller studies had reported improvements in glycaemic control and insulin sensitivity, yet a six-month, double-blind, placebo-controlled trial in 192 patients with type 2 diabetes, testing both 40 mg and 500 mg daily doses, found no significant reduction in C-reactive protein and no improvement in the broader metabolic profile relative to placebo, despite confirmed accumulation of resveratrol metabolites in plasma throughout the study. This divergence between early, smaller positive trials and later, adequately powered null trials is now a familiar pattern across the polyphenol literature, and it reinforces the central argument of this review, that bioavailability, dose, and study duration together determine whether a mechanistically plausible effect ever becomes clinically detectable [119].

### 8.2. Recent Epidemiology

Large prospective cohorts over the past decade have clarified, though not settled, how habitual polyphenol intakes relate to major outcomes. A consistent pattern emerges for mortality. In the Danish Diet, Cancer and Health cohort, total flavonoid intake showed inverse associations with all-cause, cardiovascular, and cancer mortality, with a clear plateau around ~500 mg/day and stronger gradients in smokers and heavier alcohol consumers, an observation that hints at benefit where background oxidative/inflammatory tone is higher [120].

Similar, though not identical, signals appear in other populations: a Japanese cohort linked higher total polyphenol intake (estimated with food-composition databases harmonized to Phenol-Explorer) to lower all-cause mortality, and a recent Spanish nationwide cohort reported ≈20% lower all-cause mortality across specific polyphenol subclasses at higher intakes. Taken together, these studies suggest diminishing returns above moderate daily exposures and underscore the importance of subclass resolution and population context [121].

Cardiovascular endpoints below mortality are more heterogeneous, in part because intake assessment and confounding control vary. Nonetheless, several lines point in a coherent direction. Analyses nested in contemporary cohorts describe associations between higher polyphenol or flavonoid-rich food intake and more favourable inflammatory and haemostatic profiles (for example, lower C-reactive protein and fibrinogen), providing plausible mediators for the mortality signals seen at scale [122].

These physiologic correlates also mirror effects observed in randomized trials of flavanol-rich interventions on endothelial function and blood pressure, although translation to hard events has proven elusive outside very long follow-up. Measurement is slowly improving: urinary phenyl-γ-valerolactones, colonic metabolites of flavan-3-ols, perform as objective exposure biomarkers and may help reduce misclassification in future cohort work [123].

Neuroepidemiology has advanced the most since 2020. In the Framingham Offspring cohort, higher long-term flavonoid intake was associated with lower risks of Alzheimer’s disease and related dementias, findings echoed by work on subjective cognitive decline and by studies linking flavonoid-rich fruits to lower incident dementia [124].

More recently, a “flavodiet” score aggregating flavonoid-rich foods in a large population sample associated higher scores with lower dementia risk, including in genetically susceptible participants, an approach that may be easier to translate into guidance than subclass grams. These associations do not prove causality, but their consistency across cohorts, endpoints (clinical dementia, MRI correlates, subjective decline), and assessment strategies makes a credible case for further mechanistic and interventional work [125].

Caveats remain. Residual confounding by overall diet quality and lifestyle is hard to extinguish, intake estimates rely on FFQs with limited granularity for processing and matrix effects, and interindividual “metabotypes” likely modulate exposure to active microbial metabolites in ways cohorts seldom capture. Still, the weight of evidence now supports the view that realistic, food-based intakes of polyphenols, especially flavonoids, track with lower mortality and more favourable vascular and neurocognitive trajectories over time [126].

### 8.3. Why Some Large-N Studies Show Null Effects: Dose, Adherence, Metabotypes, Matrix, and Endpoints

Null findings in large cohorts or trials rarely mean “no biology”; more often, they signal mismatches between how polyphenols work and how exposure and outcomes are measured. Dose sits at the front of the queue. Many interventions deliver amounts that are plausible but fall short of the threshold needed to nudge NRF2 targets or endothelial tone once first-pass conjugation, dilution into body water, and rapid clearance are accounted for. Duration compounds the problem: redox-signalling adaptations accrue slowly and may require weeks to remodel glutathione pools, mitochondrial quality control, or inflammatory set-points, outlasting short trials [127].

Adherence and exposure misclassification then flatten any real signal. Food-frequency questionnaires cannot resolve processing, matrix, or timing: a cup of tea is not the same exposure if brewed strong, with milk, or after storage. Even capsule trials suffer when adherence drifts or when intake is verified by aglycone assays rather than by metabolite panels that reflect what actually circulates. Interindividual metabotypes magnify heterogeneity. If only a subset efficiently generates urolithins or phenyl-γ-valerolactones, the group mean will dilute true effects unless analyses stratify by metabolite production or use objective biomarkers [128].

Matrix and co-ingestion matter more than most designs acknowledge. Protein or calcium-rich carriers can sequester reactive phenolics and defer absorption to the colon, while processing can convert recyclable diphenols into adducts that excel in chemical assays yet do little *in vivo*. Timing relative to meals, and circadian variation in transporters and phase II capacity, adds further noise. Polypharmacy is another quiet confounder: widely used drugs modulate UGT/SULT activity or gastric pH, shifting conjugation and bioaccessibility in ways few studies capture [129].

Finally, endpoints are often poorly matched to mechanism. Composite cardiovascular outcomes mix processes that move on different timescales, cognitive measures chosen for practicality may be insensitive over months, and single time-point biomarkers miss transient peroxide pulses and adaptive transcription. Nonlinear dose-response curves with plateaus mean that enrolling participants already above an effective intake will bias results to null. The remedy is not necessarily larger samples but better alignment: verify exposure with metabolite biomarkers, stratify by metabotype, respect matrix and timing, and choose endpoints that track the slow, systems-level recalibration that polyphenols most reliably induce [65].

Table 1 draws these threads together for the seven polyphenol classes discussed above, summarising circulating metabolites, physiologically achieved exposure, the experimental models used, the principal molecular targets, the current state of human clinical evidence, and the main safety observations reported to date.

## 9. From Chemistry to Clinic: A Practical Dose-Response Framework

Before we discuss specific families, we translate the mechanistic logic into a clinic-ready way of thinking about dose. Polyphenols rarely obey linear pharmacology; their actions bend into hormetic curves where small, recoverable oxidative nudges improve resilience and larger, sustained inputs erode buffers and invite futile cycling [130].

What matters, therefore, is not an abstract “antioxidant capacity” but whether a given exposure falls inside an effective window, a range bounded below by signalling thresholds (for NRF2 programs, thiol redox, endothelial tone) and above by contexts that amplify electrophile and peroxide load (labile Fe/Cu, hypoxia, GSH scarcity) [86].

Windows are family-specific because structural features set ease of oxidation, metal interactions, and quinone lifetimes, and they are person-specific because conjugation, transport, organ function, and metabotype shape circulating species. In practice, we gauge position in the window with objective biomarkers (plasma/urine conjugates and microbial metabolites, thiol indices), align matrix and timing to temper peaks, and choose endpoints that register adaptive recalibration rather than single time-point scavenging. With that scaffold, we can now map typical windows for catechols, gallates, stilbenes, and flavonols [61].

### 9.1. Hormetic Curves and “Effective Windows” by Family (Catechols, Gallics, Stilbenes, Flavonols)

Where the bend in that curve occurs is not arbitrary. It is set by a compound’s structural proclivities, its ease of oxidation, its capacity to form electrophilic quinones, and its interactions with metals, folded into the local biology of conjugation, thiol buffering, and antioxidant enzyme tone. Thinking in terms of “effective windows” is therefore more useful than speaking of generic antioxidant potency [131].

Catechols (exemplified by caffeic acid, hydroxytyrosol, and the catechol-type B-ring of epigallocatechin gallate) typically sit closest to the edge. Ortho-dihydroxylation lowers the barrier to one-electron steps, so modest concentrations can already generate the low-amplitude H_2_O_2_ transients that recruit peroxiredoxin relays and ease NRF2 activation. Push higher, or add labile iron or copper, and the same catechols tip into o-quinone formation and peroxide amplification, the window is narrow, and context, pH, oxygen, metal ligation, decides which side you see [132].

Gallates (exemplified by gallic acid, propyl gallate, and the galloyl ester of epigallocatechin gallate) behave similarly but with a twist: trihydroxylated rings and galloyl esters can donate electrons readily yet also disperse charge, sometimes widening the signalling window in protein-rich matrices that moderate metal catalysis, while tightening it in alkaline or metal-rich niches [133].

Stilbenes (exemplified by resveratrol, piceatannol, and pterostilbene, which differ from one another in methylation pattern and bioavailability) such as resveratrol occupy a slightly safer middle ground. Their phenolic pattern is less predisposed to rapid autoxidation, and much of their biology arrives through indirect routes, modest H_2_O_2_ tone, metabolic and sirtuin crosstalk, and NRF2-compatible stress responses. As a result, the window often lies at higher nominal doses, with failure more likely from poor exposure than from overt pro-oxidant drift [134].

Flavonols, quercetin foremost but also including kaempferol, myricetin, and isorhamnetin, which differ in the number and position of hydroxyl groups, straddle both behaviours. The 3-hydroxyflavone scaffold supports radical delocalization and metal chelation that can be protective at low levels, yet quercetin’s catechol B-ring makes quinone formation plausible when thiols are scarce or metals abundant. Here, the window is governed as much by host capacity, COMT activity, NQO1 induction, GSH reserves, as by the molecule itself [135].

Across families, the practical rule holds: benefits cluster where short, recoverable oxidative nudges meet intact two-electron repair and conjugation. The same intake can land inside or outside that window depending on matrix, co-ingestion, and personal biology. Designing trials, and foods, to keep typical exposures within those family-specific windows is the surest way to convert attractive chemistry into durable physiological gain [136].

It is worth stressing that even within a single family, individual compounds can differ appreciably in where their window sits, so the tendencies described above should be read as illustrative rather than as fixed values that apply uniformly to every member of a structural class.

### 9.2. Conditions That Turn an Antioxidant into a “Useful” Pro-Oxidant (Redox Preconditioning)

“Useful” pro-oxidation is less an intrinsic property of a compound than a choreography between chemistry and context. The same molecule that looks antioxidant in a tube can, in cells, provoke small, well-timed oxidative nudges that leave the system more resilient than before. Three conditions tend to make that choreography work. First, the oxidant pulse must be brief and spatially constrained [137].

H_2_O_2_ transients in microdomains, fuelled by moderate auto-oxidation of catechols near membranes or by enzyme systems already operating on the surface, reach their targets through peroxipore channels and redox relays, and are then rapidly extinguished by peroxiredoxins and glutathione peroxidases. Second, the thiol economy must be solvent, with adequate glutathione and NADPH to reverse the oxidation of the sensor cysteine and fuel NQO1-mediated two-electron repair of any quinone formed. Third, labile iron and copper must be kept tightly bound; otherwise, one-electron shortcuts turn a whisper of signalling into Fenton-driven noise [138,139].

Under these conditions, a mild pro-oxidant push becomes instructive. Transient oxidation of protein tyrosine phosphatases lifts kinase signalling above basal friction, and soft electrophiles formed in low amounts tap the Keap1 cysteine “code”, slow NRF2 turnover, and expand the cytoprotective transcriptome [140].

Downstream, the cell leans into maintenance, more glutathione synthesis and cystine import, more NQO1 and GST capacity, tighter proteostasis, and, in some settings, a nudge toward mitophagy and mitochondrial renewal that lowers basal oxidant tone. The paradox resolves: the momentary cost in oxidation is repaid by a higher set point for defence [141].

Timing and delivery matter as much as dose. Intermittent exposure favours adaptation, whereas continuous exposure erodes buffers and invites futile redox cycling. Matrix can help here: food formats that stagger liberation of reactive aglycones, or that bias metabolism toward stable microbial products (urolithins, phenyl-acids), are more likely to land within the adaptive window [142].

Ascorbate efficiently recycles quinones and can cap the pulse; amino acids and proteins mop up electrophiles but may also sequester phenolics prematurely. Finally, physiology sets the boundary conditions. Normoxia, neutral pH, and intact autophagy widen the window for preconditioning, whereas hypoxia, acidosis, inflammation, or pre-existing GSH deficit narrow it. In practice, “useful” pro-oxidation is not a license to oxidize, but a design principle: elicit small, recoverable perturbations in a system equipped to recover, and it will recover stronger [143].

### 9.3. Interactions with Drugs and Nutrients (Phase II, Transporters, Metals)

Polyphenol pharmacology is inseparable from the same systems that govern drug fate. At the biotransformation level, most flavonoids are avid substrates for Phase II conjugation, UGTs, SULTs, and, for catechols, COMT, yet they can also modulate these enzymes. *In vitro*, quercetin-, catechin-, and resveratrol-type scaffolds inhibit selected UGT and SULT isoforms at micromolar doses; at customary dietary exposures, the effect is usually muted, but concentrated supplements or co-formulations can push toward ranges where competition becomes plausible. COMT deserves a special note: catecholic polyphenols compete for the same methyl donor (S-adenosylmethionine) as endogenous catecholamines [144].

While clinically meaningful displacement is unlikely with food, high-dose extracts may shift local methylation balance in gut or liver and change the composition of metabolites that reach the circulation. Downstream, activation of NRF2 by polyphenols tends to increase expression of NQO1, GSTs, and selected conjugating enzymes, nudging the system toward faster detoxication, a generally protective tilt that nonetheless can shorten the half-life of co-administered soft electrophiles [145].

Transporters add a second, often underappreciated, layer. Intestinal uptake carriers such as OATP1A2/2B1 are sensitive to certain flavonoids and green-tea catechins, which can lower the absorption of OATP substrates when taken together [146].

Among these transporter-mediated interactions, the clearest clinical confirmation comes from green tea catechins and the beta-blocker nadolol. A controlled pharmacokinetic study in healthy volunteers showed that a single serving of green tea reduced plasma nadolol concentrations substantially, most plausibly through inhibition of intestinal OATP1A2-mediated uptake [147]. This example remains the exception rather than the rule. Most of the enzyme and transporter interactions described in this section rest on *in vitro* inhibition data or on pharmacokinetic modelling rather than on confirmed clinical outcomes, and that distinction should be kept in mind when translating these mechanisms into practical advice.

Conversely, luminal inhibition of P-glycoprotein or BCRP by some flavonols may raise the exposure of their substrates, while NRF2-driven induction of MRP2/3 favours efflux of conjugated polyphenols back into the gut or bile. Because these effects depend on timing and local concentration, patterning intake, separating a supplement or a strongly brewed beverage from critical medications, matters more than absolute dose [148].

Metal interactions are the third pillar, because many polyphenols chelate non-heme iron and, to a lesser extent, copper. In foods, this can be beneficial, blunting metal-catalysed oxidations; at the mucosal surface, it can reduce iron absorption, a predictable effect for tea and some cocoa or wine matrices when co-ingested with iron-rich meals [149].

Vitamin C counters this by reducing Fe^3+^ and promoting uptake, while proteins and calcium salts can sequester reactive polyphenols and defer their absorption to the distal gut. The same chelation chemistry also shapes safety: in metal-rich niches, weakly bound Fe/Cu can accelerate one-electron routes and narrow the redox “safety window”, whereas tight sequestration by proteins widens it [150].

These lines converge on a practical principle: interactions are real, but they depend on context. They arise at points of high local concentration (supplements, extracts, highly concentrated beverages), when taken at the same time as drugs or labile minerals, and when the redox or background conjugation capacity is compromised. In general, it is sufficient to carefully plan the timing of intake, understand the transporter substrates, and pay attention to the simultaneous intake of iron or copper to reap the benefits and avoid avoidable pharmacokinetic surprises [151].

## 10. Applications and Translation

Before translating mechanisms into practice, we need to define what, exactly, is being personalized and why. The levers available to clinicians and product developers are concrete: matrix and processing (which set quinone speciation and extractability), dose size and frequency (which govern whether exposures sit inside effective windows), timing relative to meals and exercise (which shapes proximal absorption versus colonic transformation), and co-nutrients (ascorbate, proteins, minerals) that steer redox and transport. On the receiving end, individuals differ in what they can make and clear, microbial metabotypes, Phase II capacity, transporter profiles, and renal/hepatic function together decide which species circulate and for how long [129].

Safety is contextual rather than absolute, hinging on thiol/NADPH reserves and labile metals. Effectiveness likewise depends on verified exposure rather than intake surrogates. Applications should therefore start with phenotyping (objective metabolite readouts), use food-first formats that temper peaks, and monitor mechanism-aligned biomarkers. With that scaffold, the next subsection lays out a pragmatic approach to precision nutrition: stratifying by metabotype and by basic organ and conjugation status to place real people inside family-specific effective windows [65].

### 10.1. Precision Nutrition: Stratifying by Metabotype, Renal/Hepatic Function, and Phase II Polymorphisms

Precision in the polyphenol space begins with acknowledging that people do not make, or clear, the same molecules after the same meal. The most actionable layer is metabotype. A simple post-dose “challenge” with an ellagitannin- or flavan-3-ol-rich food, followed by targeted measurement of urolithins or phenyl-γ-valerolactones in urine or plasma, cleanly separates producers from low or non-producers [62].

That information is dynamic: microbiota composition and function can shift with prebiotic fibres, fermented carriers, and longer run-in periods. For producers, modest intakes reliably cross signalling thresholds with little electrophile burden, and for non-producers, focusing on alternative sources (e.g., matrices richer in small phenolics that absorb proximally) or on strategies that enhance colonic transformation, often makes the difference between a null and a measurable response [152].

Renal and hepatic function define the next filter because the species that actually circulate are mostly conjugates. Diminished glomerular filtration slows clearance of glucuronides and sulphates, lengthening exposure and, in advanced chronic kidney disease, raising the chance that local deconjugation at inflamed interfaces regenerates aglycones where thiols are scarce [33].

Conversely, cholestasis or reduced hepatic function can blunt biliary efflux of conjugates, alter enterohepatic cycling, and change the timing of systemic peaks. In practice, a basic clinical profile, estimated GFR, liver enzymes, bilirubin, helps decide whether to favour lower, more frequent doses, to avoid concentrated extracts, or to prefer food formats that stage release and minimize sharp peaks [153].

Phase II polymorphisms and transporter genetics add finer grain. Common variants in COMT (e.g., Val158Met) shape methylation of catechols, UGT1A1*28 and other UGT/SULT haplotypes influence the balance and speed of conjugation, null or low-activity GST genotypes can lower electrophile buffering, and variation in efflux carriers (MRP2/ABCC2, BCRP/ABCG2) or uptake transporters (OATPs) modulates tissue access. Effect sizes are usually modest at dietary doses, but they matter at the margins, when high-potency supplements are used, when renal or hepatic reserve is limited, or when a person’s clinical phenotype suggests unusually fast or slow clearance [54].

We set out this sequencing here as a research priority rather than as validated clinical guidance, since none of these strategies has yet been tested in a prospective trial that randomises people to phenotype-guided or genotype-guided dosing and follows clinical outcomes. A pragmatic workflow follows from these layers: phenotype first, genotype when needed. Verify exposure with metabolite biomarkers after a representative meal, adapt matrix and timing to the person’s metabotype and organ function, and reserve genotyping for unexplained non-response, adverse effects, or research settings. Testing this workflow directly, to establish whether phenotype-first stratification actually changes outcomes, should precede any recommendation for routine clinical use. This sequencing respects how polyphenols work *in vivo*, through regulated formation and handling of metabolites, while giving clinicians and researchers levers to move individuals into the effective window with minimal oxidative “spend” [154].

### 10.2. Food/Ingredient Design: Modulating PPO and Quinone Speciation

Designing polyphenol-rich foods is, in essence, an exercise in steering quinone traffic. The same processing steps that make products safe, stable, and palatable also decide whether phenolics remain as reversible diphenols or are driven into long-lived adducts and pigments. A first principle is early control of polyphenol oxidase (PPO). Rapid heat treatments that denature the dicopper active site, mild acidification toward the enzyme’s suboptimal range, and tight management of dissolved oxygen arrest the initial burst of o-quinone formation after tissue disruption [93].

Where “fresh-like” processing is required, the combination of low-oxygen handling, brief pressure or thermal pulses, and chelation of trace Fe/Cu can suppress radical side routes without materially altering flavour. Reducing agents such as ascorbate efficiently recycle quinones to diphenols but should be dosed with restraint, as they inflate electron-transfer assay readouts and can mask underlying losses of tractable catechols [96].

A second principle is kinetic partitioning. If o-quinones are allowed to form, their fate can be guided toward benign sinks. Protein-rich carriers, added cysteine equivalents, or yeast-derived glutathione cap electrophiles rapidly, limiting random adduction to native proteins and confining polymerization. This approach trades a fraction of free phenolics for a controlled adduct pool with less sensory impact, it is especially useful in matrices where astringency must be tempered [155].

By contrast, extensive sulphiting, where permitted, “bleaches” quinones at the cost of erasing electrophile-driven signalling and is increasingly avoided. Process water activity and pH modulate all of these flows: lower a_w slows both PPO turnover and non-enzymatic coupling, while modest acidification reduces phenolate formation and stabilizes diphenols during storage [104].

Microstructure and delivery complete the design. Encapsulation in biopolymer matrices, emulsification into lipid phases, or retention within partially intact cell walls slows oxygen access and staggers release during digestion, preserving a larger share of reversible redox couples to the point of absorption [156].

Fermentation can be harnessed deliberately: microbial glycosidases liberate aglycones, while selected starters limit laccase-like activities that would otherwise push chemistry toward inert pigments. In cocoa- and tea-like systems, measured oxidation can be used to build target dimers (e.g., theaflavins) while stopping short of deep polymerization [157].

Finally, packaging and headspace are not afterthoughts: deaeration before bottling, oxygen-impermeable films, and modified atmospheres keep quinone speciation within a reversible envelope. The unifying aim is pragmatic rather than doctrinal, maximize the fraction of phenolics that can recycle between diphenol and quinone under physiological conditions, minimize irreversible coupling, and deliver those species in formats that respect both sensory quality and the biology of redox signalling [158].

### 10.3. Opportunities in Healthy Ageing, Muscle, and Brain

If healthy ageing is the art of preserving reserve, metabolic, vascular, and neural, then polyphenols fit best not as blunt antioxidants but as gentle tutors of resilience. Their value lies in nudging stress-response circuits toward readiness while keeping oxidative “spend” low. By tilting the Keap1, NRF2 axis toward inducibility, improving mitochondrial housekeeping, and softening inflammatory tone, they help set a cellular milieu in which everyday insults are handled more efficiently. That recalibration is most apparent when background risk is nontrivial: older adults with low-grade inflammation, diminished microvascular function, or early declines in muscle performance and cognition [159].

Skeletal muscle offers a clear test bed. With age, mitochondrial turnover slows, capillary responsiveness blunts, and redox buffers thin, conditions under which small, recoverable oxidative cues are unusually effective. Diets that deliver stable microbial metabolites (such as urolithin A) or modest peaks of proximal absorbers (such as epicatechin-rich flavanols) can improve the quality of the oxidative milieu in which muscle contracts and adapts [160].

The practical opportunity is to pair intake with exercise, using training as the primary stimulus and polyphenols to lower the friction: better endothelial nitric oxide signalling, faster recovery of thiol balance, and a quieter inflammatory aftermath. In pre-frail or sedentary older adults, this “exer-nutrition” approach may convert otherwise subthreshold training into measurable gains in strength, gait speed, or fatigue resistance without resorting to pharmacologic doses [161].

In the brain, the same principles scale to neurovascular and glial biology. Cognitive ageing is as much about blood flow and microglial set-points as it is about neurons. Flavanol-rich foods that improve endothelial responsiveness can sharpen neurovascular coupling, while polyphenol-derived signals that favour NRF2 in astrocytes and microglia help maintain redox housekeeping and limit sterile inflammation [162].

Here, the gut-brain axis is not a slogan but a delivery route: phenyl-γ-valerolactones and selected phenyl-acids circulate for hours at low micromolar levels and can subtly adjust inflammatory and vascular tone in a way that aligns with preserved executive function and memory over time. None of this obviates the need for sleep, cognitive engagement, or cardiovascular risk control, it simply lowers the biologic threshold at which those investments pay off [163].

Translating these ideas is less about finding “the” superfood than about design. Small, frequent doses embedded in meals, matrices that curb PPO-driven losses and stagger release, and timing relative to exercise or cognitively demanding periods are all levers that keep exposure inside the effective window. Stratifying by metabotype and renal function improves signal detection and safety, while tracking objective metabolites alongside functional endpoints keeps programs honest [164].

The scientific horizon is equally practical: longer trials that target pre-frailty and subjective cognitive decline, factorial designs that combine exercise with tailored polyphenol delivery, and food formats that privilege reversible redox couples over inert pigments. In short, the opportunity is to use polyphenols not to smother oxidants, but to coach systems, muscle and brain foremost, into a steadier, more youthful equilibrium [165].

## 11. Gaps, Controversies, and a 2025–2030 Agenda

Before we lay out a research agenda, we need to untie the conceptual knots that keep the field from accumulating cleanly. Chief among them is vocabulary. “Antioxidant” migrated from food chemistry to physiology carrying assumptions that do not survive contact with cells, yet simply rebranding polyphenols as “redox signalling agents” risks swapping one imprecision for another. The tension is sharpened by measurement: cuvette assays reward electron transfer under artificial conditions, while the species that circulate *in vivo*, conjugates and microbial metabolites, act primarily by nudging peroxide relays and electrophile sensors, not by mopping up radicals [166].

Add to this the practical pressures of marketing and regulation, which favour simple claims and discourage talk of hormesis or context, and we get a literature that labels the same molecule antioxidant or pro-oxidant depending on where and when it is tested. Our stance in what follows is deliberately operational: terms should be tied to matrix, dose, timing, and readout, and defended with biomarker evidence rather than surrogates. With that in view, we first examine the definitional dispute “antioxidant” versus “signalling agent”, and what would count as a fair, falsifiable use of each term.

### 11.1. Conceptual Controversies (Definitions, “Antioxidant” vs. “Signalling Agent”)

Much of the confusion in the polyphenol field stems from language that predates our present grasp of redox biology. “Antioxidant” was once a serviceable umbrella for molecules that slowed autoxidation in foods or quenched radicals *in vitro*. Transplanted into physiology, the term becomes slippery. It conflates fundamentally different actions, direct electron transfer, metal chelation, peroxide scavenging, induction of endogenous defences, and erases the fact that the same compound can look protective in one matrix and injurious in another. The result is a literature that swaps labels as context shifts: the very catechol that “prevents oxidation” at low dose becomes “pro-oxidant” in metal-rich media, even though the underlying chemistry has not changed [167].

A second fault line is measurement. Chemical assays such as DPPH, FRAP, ORAC, and CUPRAC are frequently treated as portable proxies for biological benefit. They are not. They quantify electron-transfer capacity or radical quenching under assay conditions that rarely resemble a cell, let alone a tissue. When these numbers are promoted to mechanistic claims, we misread signal as substance and set trials up to look for effects that the *in vivo* species, mostly conjugates and microbial metabolites, are not designed to deliver [168].

The proposed alternative, calling polyphenols “redox signalling agents”, carries its own risks. Critics worry that the term romanticizes mild pro-oxidant behaviour and underplays hazards from quinone formation and thiol adduction. Proponents counter that signalling is empirically how benefits arise: small, compartmentalized peroxide transients and soft-electrophile nudges that bias Keap1-NRF2, proteostasis, and mitochondrial maintenance toward readiness [169].

The resolution lies in precision, not polemic. “Antioxidant” and “pro-oxidant” should be treated as operational descriptors tied to a matrix, dose, timescale, and readout; “signalling agent” should never be a free pass, but a hypothesis about mechanism that invites falsification with appropriate biomarkers (NRF2 targets, thiol redox, peroxiredoxin states), not with cuvette colour changes [170].

As a community, we can do better: define terms explicitly, report conditions that set the sign (pH, O_2_, metals, protein and lipid context), prioritize exposure biomarkers over intake surrogates, and choose endpoints that align with slow, adaptive recalibration. If we retire “antioxidant” as a catch-all and reserve it for clearly specified outcomes, much of the paradox dissolves [171].

### 11.2. Open Questions (Non-Responders, Chronobiology, Microbiota)

“Non-responders” remain the field’s most vexing category, and it is not clear how often they are truly unresponsive versus simply misclassified. Some people may never reach effective exposures because conjugation is brisk, transporters return conjugates to the lumen, or renal clearance is high, while others may respond through pathways we do not measure [172].

The practical question is where the failure lies: delivery, chemistry, or readout. Short, standardized “challenge” tests with objective metabolites (e.g., urolithins, phenyl-γ-valerolactones) could separate exposure failure from biology failure, yet they are rarely embedded in trials. Equally unresolved is how organ reserve, mild renal impairment, subclinical cholestasis, low glutathione tone, shifts windows of benefit and risk at habitual intakes. Genotype likely contributes at the margins (COMT, UGT, SULT, GST, transporters), but we still lack a hierarchy that tells us when genotyping changes practice and when phenotyping is enough [173].

Chronobiology is an even larger blind spot. Phase II enzymes, thiol pools, and hepatic and intestinal transporters oscillate across the day, as do peroxiredoxin cycles and NRF2 target readiness. Feeding-fasting transitions, bile acid rhythms, gastric emptying, and colonic motility gate when and where aglycones or microbial metabolites appear [174].

We do not know whether morning versus evening dosing changes the balance between proximal absorption and colonic transformation, or whether aligning intake with exercise or cognitively demanding periods improves signal-to-noise for performance and neurocognitive endpoints. Trials seldom control for time-of-day, yet the mechanisms we invoke, redox relays, Keap1 sensing, endothelial tone, are time-dependent by design. Establishing whether circadian alignment matters is low-hanging fruit with high translational yield [175].

The microbiota layer raises questions of causality and durability. Metabotypes are reproducible within individuals, but we do not know how stable they are over months, what minimal dietary or prebiotic “dose” flips a non-producer into a producer, or whether targeted starters can be used safely and persistently in real foods [176].

We also lack clarity on where the action sits: luminal metabolites measured in stool do not necessarily reflect mucosal chemistry, where β-glucuronidases and sulphatases regenerate aglycones at inflamed interfaces. How often does local deconjugation, rather than systemic microbes, carry the adaptive signal? And which guilds, not single species, are functionally essential for each pathway [177]?

Answering these questions will require designs that move beyond static snapshots: repeated-measures, time-stamped biomarker panels around standardized challenges, factorial crossovers that test dose and dosing time, and microbiota interventions evaluated for function, not just composition. Until then, “non-response”, “optimal timing”, and “the microbiome effect” will remain labels for uncertainty rather than settled biology [178].

### 11.3. Shared Resources: Physicochemical Data, Scripts, and Mechanistic Maps

If the field is to move beyond slogans and inconsistent proxies, we need common scaffolding that lets different groups ask comparable questions and obtain commensurable answers. Three layers are worth building as “living” resources: a rigorously curated physicochemical dataset, a suite of reproducible analysis scripts, and an interactive mechanistic map that ties chemistry to biology and processing [179].

The physicochemical layer should do more than list names and structures. For each archetypal polyphenol (and, where relevant, key microbial metabolites), it should report pKa values for each ionizable site, standard reduction potentials referenced to NHE, O-H bond dissociation enthalpies, logP/logD (including pH 7.4), and stability constants for Fe/Cu complexes under defined ionic strength [180].

Kinetic descriptors belong here too: apparent autoxidation rates at controlled pH/O_2_, second-order rate constants for reaction with glutathione, and summary kinetic parameters for NQO1 repair and common UGT/SULT reactions. Every entry should carry provenance and uncertainty, with machine-readable identifiers (SMILES, InChIKey) and crosswalks to major databases, so the table can serve as both citation and substrate for modelling [181].

On top of that, shared scripts, not notebooks as afterthoughts, but versioned tools, can turn static numbers into insight: e.g., speciation calculators that propagate pH and ionic strength into phenolate fractions, simulators that estimate H_2_O_2_ flux from autoxidation under variable O_2_ and trace metals, routines that fit hormetic dose-response curves and return “adaptive window” metrics with confidence bands, pipelines that parse LC-MS/MS data for urolithins, phenyl-γ-valerolactones, and conjugates with automated QC, and harmonizers that translate food-frequency data into subclass intakes using transparent composition mappings. These should ship with unit tests, containerized environments, and example datasets so that a lab can reproduce figures and tables from the manuscript with a single command [182].

Finally, an interactive mechanistic map can make the framework teachable and testable. Nodes would include polyphenol families, quinone species, enzymes (NQO1, GST, UGT/SULT, COMT, PPO), transporters (MRPs, BCRP, OATPs), redox relays (peroxiredoxins), sensors (Keap1, PTPs), and oxidases (XO, MAO, COX-2), with edges annotated as “generates H_2_O_2_”, “consumes GSH”, “activates”, or “inhibits”, each backed by an evidence link. Contextual overlays, pH, oxygen tension, labile metals, hypoxia, microbiota metabotypes, processing steps, would let users visualize how speciation and signalling shift across matrices or individuals, and export testable predictions for trials or product design [183].

All three layers should be openly licensed (e.g., CC-BY for data, MIT/Apache for code), archived with DOIs at each release, and accompanied by a minimal reporting checklist (pH, O_2_, metal speciation, protein/ascorbate context, enzyme activities, timing). If we lower the friction to reuse and scrutiny, we also raise the odds that “polyphenol science” becomes quantitative, comparable, and, ultimately, more clinically useful [184].

## 12. Conclusions

The past decade has made it hard to sustain the old, catch-all language of “antioxidants”. What emerges instead is a tractable picture of polyphenols as modulators of redox signalling whose biological sign, protective or injurious, depends on context. We have framed this with operational definitions and a concrete mechanism: catechol and hydroquinone motifs that can climb to o- and p-quinones, and an enzyme-guarded loop, dominated by NQO1, glutathione systems, and conjugation pathways, that either repairs that chemistry or lets it spiral into futile cycling. Low, localized hydrogen-peroxide transients, ferried across membranes by peroxiporins and decoded by redox relays, couple this chemistry to protein sensors such as Keap1 and to phosphatases that set signalling thresholds. When inputs are brief and buffers are intact, the net result is hormetic: stronger endogenous defences at little oxidative “spend”.

This logic travels from bench to plate to clinic. In foods, PPO and processing steer quinone speciation, and the balance between reversible redox couples and terminal pigments, matrices, pH, oxygen, and trace metals all move the needle. *In vivo*, first-pass conjugation and microbial biotransformation mean that conjugates, urolithins, and phenyl-acids, not parent aglycones, carry much of the signal, with distinct exposure profiles across metabotypes. Human evidence is no longer anecdotal: large trials and cohorts suggest vascular and neurocognitive benefits under realistic intakes, yet they also expose why null results occur, doses below effective windows, exposure misclassification, insensitive endpoints, and unmeasured heterogeneity.

The practical message is straightforward. Claims and designs should be tied to matrix, dose, timing, and readout, exposure should be verified with metabolite biomarkers rather than inferred from intake, participants should be stratified by metabotype and basic organ function, and endpoints should reflect slow, systems-level recalibration (NRF2 programs, thiol redox, endothelial function) rather than single time-point scavenging assays. On the research side, a common scaffold, open physicochemical data, reproducible scripts, and mechanistic maps, can turn scattered findings into cumulative knowledge.

Recast this way, polyphenols are neither panaceas nor paradoxes. They are tuneable inputs to a conserved stress-response network. Our task is to keep exposures inside family-specific effective windows, to report the conditions that set the sign, and to measure what matters. Do that, and the field moves from slogans to solutions.

## Figures and Tables

**Table 1:** Summary of major polyphenol classes discussed in this review, their circulating metabolites, physiological exposure, experimental models, molecular targets, human clinical evidence, and safety notes.

Polyphenol Class (Representative Compound)	Main Circulating Metabolites	Physiological Exposure Range	Experimental Models Used	Key Molecular Targets	Human Clinical Evidence (2015–2025)	Safety Notes
Cocoa flavan-3-ols (epicatechin)	Epicatechin glucuronides and sulphates, phenyl-γ-valerolactones	Low micromolar range in plasma after realistic intakes	Endothelial cell assays, large randomised trials (COSMOS)	eNOS, NRF2, inflammatory markers such as hsCRP	COSMOS reduced cardiovascular mortality but not the primary composite endpoint, no cognitive benefit in COSMOS-Mind	Well tolerated across trial durations of several years, no safety signal reported
Green tea catechins (EGCG)	EGCG glucuronides and sulphates, limited free aglycone	Sub-micromolar to low micromolar plasma concentrations	Prostate epithelial models, twelve-month randomised trial	NF-κB, proteasome activity, phase II detoxifying enzymes	Null effect on the primary composite prostate cancer endpoint, significant reduction on a narrower secondary endpoint	Well tolerated at 400 mg EGCG per day, routine hepatic monitoring advised at higher doses
Anthocyanins (cyanidin and delphinidin glycosides)	Phenolic acids and glucuronides generated mainly by colonic microbiota	Low nanomolar parent compound, higher for microbial phenolic acids	Endothelial function studies, six-month randomised trial in metabolic syndrome	Endothelial nitric oxide signalling, inflammatory and lipid biomarkers	Improved cardiometabolic biomarkers over 6 months, effect size modest and metabotype-dependent	Good tolerability reported at food-based intakes
Flavonols (quercetin)	Quercetin glucuronides, sulphates, and isorhamnetin conjugates	Low micromolar range achievable at supplemental doses	Vascular smooth muscle studies, randomised trials in hypertensive adults	NADPH oxidase, endothelial nitric oxide synthase	Meta-analysis of seven trials shows consistent systolic and diastolic blood pressure reduction, greater above 500 mg per day	Generally well tolerated, mild gastrointestinal effects at high doses
Stilbenes (resveratrol)	Resveratrol glucuronides and sulphates, very low free aglycone	Very low nanomolar free compound despite gram-level intakes	SIRT1 and NRF2 cell models, six-month randomised trial in type 2 diabetes	SIRT1, NQO1, inflammatory pathways	Early small trials suggested glycaemic benefit, a well powered six-month trial found no effect on CRP or metabolic profile	Well tolerated, mild gastrointestinal effects reported at gram-level doses
Ellagitannin-derived urolithins (urolithin A)	Urolithin A and B glucuronides, production strongly metabotype-dependent	Highly variable between individuals according to microbial metabotype	Muscle and mitochondrial studies, randomised trials in older and middle-aged adults	Mitophagy pathways, mitochondrial biogenesis markers	Improved muscle endurance and mitochondrial biomarkers, primary performance endpoints not always significant	Good tolerability across studies, no serious adverse events reported

## Data Availability

No new data were created or analysed in this study. Data sharing is not applicable to this article.
